# A neuronal action of sirtuin 1 suppresses bone mass in young and aging mice

**DOI:** 10.1172/JCI152868

**Published:** 2022-12-01

**Authors:** Na Luo, Ioanna Mosialou, Mattia Capulli, Brygida Bisikirska, Chyuan-Sheng Lin, Yung-yu Huang, Peter T. Shyu, X. Edward Guo, Aris Economides, J. John Mann, Stavroula Kousteni

**Affiliations:** 1 Department of Genetics and Development and; 2 Department of Physiology and Cellular Biophysics, Columbia University Medical Center, New York, New York, USA.; 3 Department of Biotechnological and Applied Clinical Sciences, University of L’Aquila, L’Aquila, Italy.; 4 Department of Pathology and Cell Biology and; 5 Department of Psychiatry, Columbia University Medical Center, New York, New York, USA.; 6 Molecular Imaging and Neuropathology Area, New York State Psychiatric Institute, New York, New York, USA.; 7 Deparment of Biomedical Engineering, Columbia University, New York, New York, USA.; 8 Regeneron Pharmaceuticals Inc., Tarrytown, New York, USA.; 9 Department of Radiology, Columbia University Medical Center, New York, New York, USA.

**Keywords:** Bone Biology, Neuroscience, Mouse models, Neuroendocrine regulation, Osteoporosis

## Abstract

The various functions of the skeleton are influenced by extracellular cues, hormones, and neurotransmitters. One type of neuronal regulation favors bone mass accrual by inhibiting sympathetic nervous system (SNS) activity. This observation raises questions about the transcriptional mechanisms regulating catecholamine synthesis. Using a combination of genetic and pharmacological studies, we found that the histone deacetylase sirtuin 1 (SIRT1) is a transcriptional modulator of the neuronal control of bone mass. Neuronal SIRT1 reduced bone mass by increasing SNS signaling. SIRT1 did so by increasing expression of monoamine oxidase A (*MAO-A*), a SIRT1 target that reduces brain serotonin levels by inducing its catabolism and by suppressing tryptophan hydroxylase 2 (*Tph2*) expression and serotonin synthesis in the brain stem. SIRT1 upregulated brain catecholamine synthesis indirectly through serotonin, but did not directly affect dopamine β hydroxylase (*Dbh*) expression in the locus coeruleus. These results help us to understand skeletal changes associated with selective serotonin reuptake inhibitors (SSRIs) and may have implications for treating skeletal and metabolic diseases.

## Introduction

Aging, as well as the pathogenesis of degenerative diseases, is intimately linked with progressive organ decline. An emerging theme in the study of the pathogenesis of age-related diseases is that organs are not silos acting independently of each other to fulfill their specific functions, but act in a coordinated and interdependent manner to regulate physiological functions. This implies that dysfunction of an aging organ may affect the function of other organs. This applies also to the skeleton. Testing this concept of functional connections between organs has gained ground over the last 15 years, with growth of our understanding of the molecular basis of skeletal physiology. Often, these advances involved discovering a role in bone remodeling for molecules already known to fulfill different functions in other organs. Three examples that illustrate this point are the emergence of RANKL, previously recognized to be a regulator of lymphocyte biology, as a major regulator of osteoclast differentiation (1); of osteocalcin and lipocalin 2 as determinants of various endocrine functions (2–10); and of leptin, an adipokine suppressing appetite and energy expenditure, as a critical regulator of the bone remodeling cycle (2). The discovery of leptin-dependent regulation of bone mass led to the discovery of the central control of bone mass and the role of the sympathetic nervous system (SNS) in this pathway. Leptin functions indirectly through the SNS to inhibit bone mass accrual. In turn, catecholamines signal through the β2-adrenergic receptor (Adrβ2) present in osteoblasts to suppress bone formation and favor bone resorption (3, 4). This brain-dependent regulation of bone mass identified in mice has been subsequently verified in humans, sheep, and rats (5, 6).

The SNS regulation of bone mass is conserved in humans, as patients on beta blockers have increased bone mineral density (BMD) and perhaps reduced fracture risk (7–10), and, more recently, it has emerged as a promising new treatment for osteoporosis (11). Plasma norepinephrine (NE) levels increase with age (12–14) and could contribute to age-related changes in bone remodeling via increased sympathetic signaling in osteoblasts, a negative regulator of bone formation and an activator of osteoblast-mediated bone resorption. Therefore, deciphering the molecular mechanisms underpinning this interaction between the brain and bone can have a major clinical impact. We chose to examine transcriptional modulators.

In invertebrates and in vertebrates, antiaging mechanisms benefit cell function in many tissues and are often facilitated by a transcriptional regulator, sirtuin 1 (SIRT1). Sirtuins are a family of nicotinamide adenine dinucleotide (NAD) — dependent proteins that deacetylate transcriptional regulators or enzymes involved in cell metabolism. Mammals have 7 sirtuins, which perform nonredundant functions in adapting physiology to environmental and metabolic stress, such as food scarcity, with implications for aging-associated diseases (15–17). In fact, the overarching hypothesis regarding sirtuin functions is that their activity matches cellular physiology to energy availability (18, 19). To achieve this purpose, sirtuins, and chief among them SIRT1, integrate intracellular networks and intercellular signaling to facilitate interorgan communications in all species tested. In model organisms, forced expression of the sirtuin homologue increases metabolic efficiency and extends life span. Similarly, in mammals, SIRT1 gain-of-function primes the organism for metabolic adaptation to insulin resistance by increasing hepatic insulin sensitivity and decreasing whole-body energy requirements. In addition, SIRT1 is involved in mitochondrial biogenesis and cellular redox. Interestingly, studies by others have shown that SIRT1 promotes osteoblast and suppresses osteoclast numbers (20–23). *Sirt1* is broadly expressed in brain (24) and, similarly to SNS activity, its levels also increase with aging (25). These observations prompted us to determine whether, in addition to its peripheral actions, SIRT1 acts indirectly to affect bone cells by modulating SNS activity.

We have identified an additional mode of action for SIRT1 in bone and delineated in vivo the molecular mechanism that mediates it. This action occurs independently of direct effects of SIRT1 peripherally in osteoblasts and osteoclasts. Instead, it results from the ability of neuronal SIRT1 to suppress serotonin synthesis and increase serotonin catabolism in the brain, subsequently leading to an increase in SNS activity. *Sirt1* inactivation in serotonergic neurons and globally in brain increases serotonin levels and suppresses catecholamine levels, diminishing SNS signaling, and as a result, increases bone mass.

## Results

### Increased sympathetic tone and decreased bone mass in Sirt1 transgenic mice.

**Figure 1:**
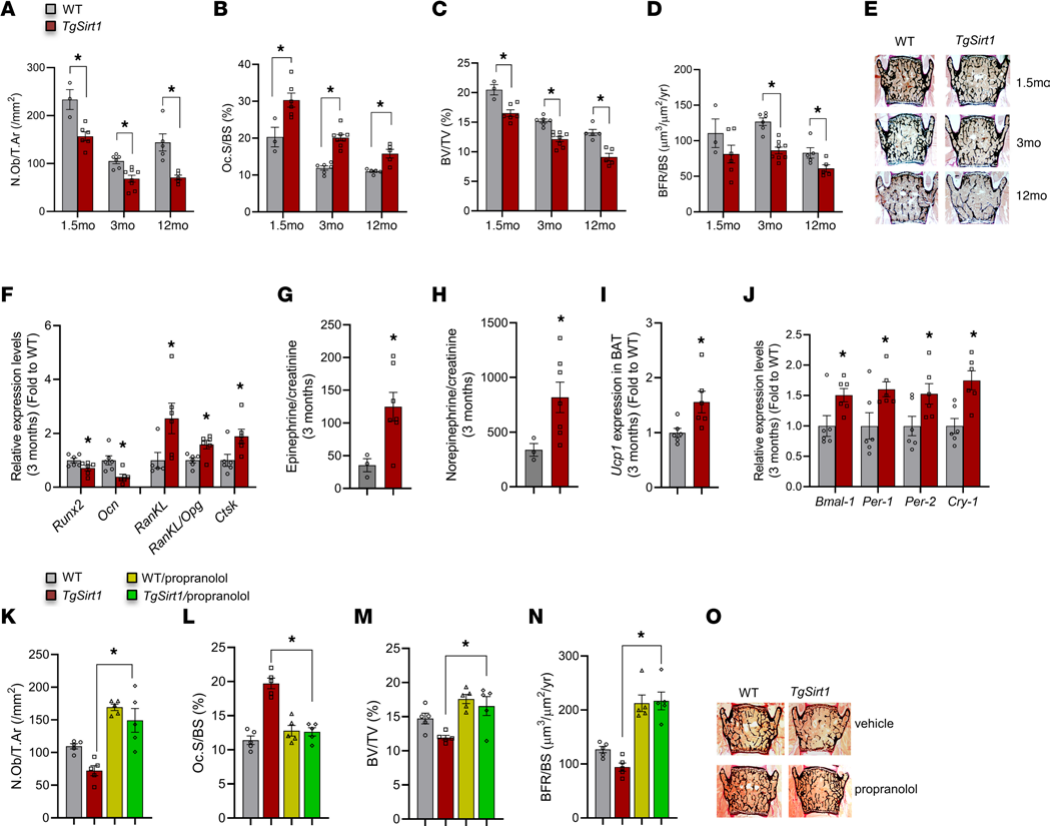
Increased sympathetic tone and decreased bone mass in *TgSirt1* mice. (**A**) Number of osteoblasts per trabecular area (N.Ob/T.Ar) (/mm^2^); (**B**) osteoclast surface per bone surface (Oc.S/BS) (%); (**C**) bone volume over tissue volume (BV/TV) (%); and (**D**) BFR/BS (μm^3^/μm^2^/yr) of *TgSirt1* mice (1.5 months: *n* = 6; 3 months: *n* = 8; 12 months: *n* = 5) versus WT controls (1.5 months: *n* = 3; 3 months: *n* = 6; 12 months: *n* = 5) at 1.5, 3, and 12 months of age. (**E**) Representative images of spines from *TgSirt1* and WT control mice stained with von Kossa. (**F**) Relative expression levels of osteoblast and osteoclast differentiation marker genes in long bones of 3-month-old *TgSirt1* mice (*n* = 6) versus WT controls (*n* = 6). (**G**) Urine epinephrine levels in 3-month-old *TgSirt1* mice (*n* = 7) versus WT controls (*n* = 3). (**H**) Urine NE levels in 3-month-old *TgSirt1* mice (*n* = 7) versus WT controls (*n* = 3). (**I**) *Ucp1* expression levels in BAT of 3-month-old *TgSirt1* mice (*n* = 6) versus WT controls (*n* = 6). (**J**) Relative expression levels of sympathetic tone target genes in long bones of 3-month-old *TgSirt1* mice (*n* = 6) versus WT controls (*n* = 6). (**K**) N.Ob/T.Ar (/mm^2^); (**L**) Oc.S/BS (%); (**M**) BV/TV (%); and (**N**) BFR/BS (μm^3^/μm^2^/yr) of 3-month-old *TgSirt1* and WT mice treated with propranolol (WT: *n* = 5; *TgSirt1*: *n* = 5; WT/propranolol: *n* = 5; *TgSirt1*/propranolol: *n* = 5). (**O**) Representative images of spines from 3-month-old *TgSirt1* and WT mice treated with propranolol stained with von Kossa. Data are represented as mean ± SEM. (**A**–**J**) *P < 0.05, *TgSirt1* versus WT by Student’s *t* test. (**K**–**N**) *P < 0.05, *TgSirt1* treated with propranolol versus *TgSirt1* by 1-way ANOVA.

As a first approach to test whether and how SIRT1 regulates bone remodeling, we used bacterial artificial chromosome (BAC) transgenic mice with moderate overexpression of *Sirt1*, equivalent to one extra gene copy (26). Increasing expression of *Sirt1* in *TgSirt1* mice moderately but ubiquitously delivers a dual compromising signal to the skeleton by concomitantly suppressing osteoblasts (Figure 1A) and increasing osteoclast numbers (Figure 1B), resulting in decreased bone volume (Figure 1, C and E) and bone-formation rate (BFR) (Figure 1D). The same magnitude of decrease in bone volume and changes in osteoblast and osteoclast numbers was maintained during aging in *TgSirt1* mice as compared with that in same-age WT littermates (Figure 1, A–E). Consistent with these cellular changes, expression of osteoblast differentiation genes (*Runx2* and osteocalcin) was decreased, whereas expression of genes favoring osteoclastogenesis, cathepsin K (*Ctsk*) and *RankL* as well as the *RankL/Opg* mRNA ratio, were increased in the bone of *TgSirt1* mice (Figure 1F). This opposite action on bone formation and bone resorption is reminiscent of the one conferred by the SNS (3, 4). Therefore, we determined whether SNS activity was increased in *TgSirt1* mice. We found that urinary epinephrine and NE as well as expression of *Ucp1* in brown adipose tissue (BAT) were increased in *TgSirt1* mice (Figure 1, G–I).

SNS outflow on bone consists of release of NE, which acts on Adrβ2 on osteoblasts to increase the expression of clock genes *BmaI1*, *Per1*, *Per2*, and *Cry1* and also works by activating osteoclasts through increasing the expression of *RankL* in osteoblasts (27–29). Therefore, besides measurement of *Ucp1* expression in BAT or catecholamine levels in urine, SNS outflow on the skeleton was determined by assessment of SNS clock target and *RankL* gene expression in bone. Indeed, expression of *BmaI1*, *Per1*, *Per2*, *Cry1*, and *RankL* was increased in the bone of *TgSirt1* mice (Figure 1, F and J). Although bone NE levels were not measured, and this could be a limitation, these results support our hypothesis that *Sirt1* overexpression in *TgSirt1* mice increases SNS signaling in osteoblasts.

### Inhibition of the sympathetic tone normalizes bone mass in TgSirt1 mice.

Next, we determined whether upregulation of SNS signaling could explain the low bone mass phenotype of *TgSirt1* mice. For this purpose, we treated *TgSirt1* mice and WT littermates with propranolol, a beta blocker, in a dose reported to block Adrβ2 signaling in bone (4). Propranolol rescued both the decrease in osteoblast numbers (Figure 1K) and the increase in osteoclast numbers (Figure 1L) in *TgSirt1* mice. BFR and bone volume were similar to those of WT controls (Figures 1, M–O). As expected, propranolol increased bone mass in WT mice (Figure 1, K–O, and refs. 4, 30).

### Sirt1 inactivation in the brain decreases sympathetic tone and increases bone mass.

**Figure 2:**
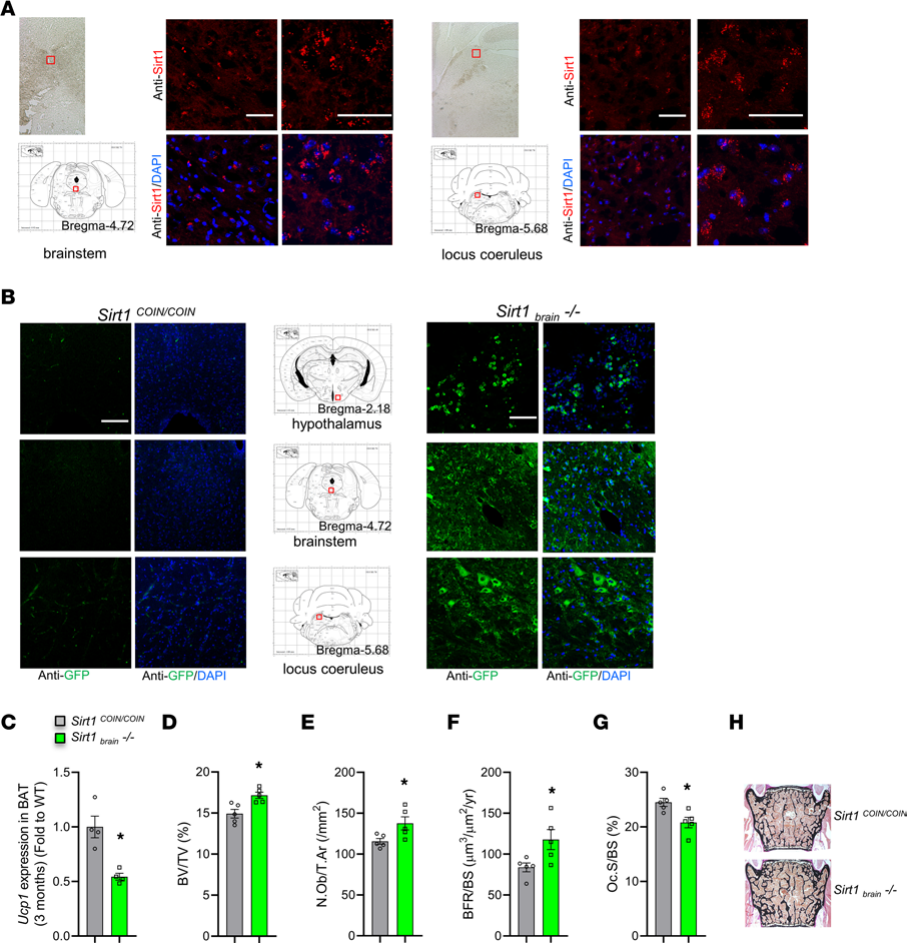
Neuronal SIRT1 regulates SNS activity and controls bone mass. (**A**) SIRT1 immunostaining in brain sections of WT mice including brain stem (left panel) and locus coeruleus (right panel). Scale bars: 100 μm. Bright field images on the left demonstrate the region of the brain under study and the coordinates in mouse brain atlas. (**B**) GFP immunostaining in hypothalamus, brain stem, and locus coeruleus sections of Adeno-CMV-Cre i.c.v. injected *Sirt1^COIN/COIN^* (*Sirt1_brain_^–/–^*) mice. Scale bars: 100 μm. (**C**) *Ucp1* expression levels in BAT of Adeno-CMV-Cre i.c.v. injected 3-month-old *Sirt1^COIN/COIN^* (*Sirt1_brain_^–/–^*) mice (*n* = 4) versus *Sirt1^COIN/COIN^* controls (*n* = 4). (**D**) BV/TV (%); (**E**) N.Ob/T.Ar (/mm^2^); (**F**) BFR/BS (μm^3^/μm^2^/yr); and (**G**) Oc.S/BS (%) of 3-month-old *Sirt1_brain_^–/–^* mice (*n* = 5) versus *Sirt1^COIN/COIN^* controls (*n* = 5). (**H**) Representative images of spines from 3-month-old *Sirt1_brain_^–/–^* mice versus *Sirt1^COIN/COIN^* controls stained with von Kossa. Data are represented as mean ± SEM. *P < 0.05 versus *Sirt1^COIN/COIN^* by Student’s *t* test.

*Sirt1* is broadly expressed in brain, including in serotonergic neurons (24), suggesting that the high sympathetic tone in *TgSirt1* mice may be due to a central effect of SIRT1. To examine this hypothesis, we followed two approaches. First, we examined *Sirt1* expression in the brain. Immunofluorescence analysis in coronal brain sections showed that *Sirt1* is also expressed in the brain stem and locus coeruleus, both areas regulating SNS activity (Figure 2A). Subsequently, we generated mice carrying a conditional null allele of *Sirt1* to determine whether neuronally expressed *Sirt1* can regulate bone mass (Supplemental Figure 1, A and B; supplemental material available online with this article; https://doi.org/10.1172/JCI152868DS1). Exon 3 carries the catalytic domain of *Sirt1*, and its deletion effectively silences *Sirt1* expression (24). A conditional null allele of *Sirt1* with EGFP as a post-Cre reporter was generated using the method conditional by inversion (COIN) (31–33).

As a first step, we performed adenovirus-mediated deletion of *Sirt1* in the brain. This was achieved by injecting 3-month-old *Sirt1^COIN/COIN^* mice in the third ventricle with an adenovirus expressing recombinant *Cre* (Adeno-CMV-Cre). *Sirt1* was efficiently inactivated in the entire brain, as shown by recombination PCR and GFP immunostaining in the hypothalamus, brain stem, locus coeruleus, and the remaining parts of the brain (Figure 2B and Supplemental Figure 1C). We found that *Sirt1* inactivation in the brain decreased SNS activity, which was shown by the decrease in *Ucp1* expression in BAT in these mice (Figure 2C), and increased bone mass by increasing bone formation and suppressing bone resorption (Figure 2, D–H). To demonstrate a direct cause and effect relationship between decreased SNS activity and increased bone mass following inactivation of *Sirt1* in the brain, we injected Adeno-CMV-Cre (or empty vector) in the third ventricle of 3-month-old *Sirt1^COIN/COIN^* male mice and treated both groups with vehicle or propranolol. We found that inhibition of SNS output in osteoblast by propranolol does not further increase bone mass in mice lacking *Sirt1* in the brain (Supplemental Figure 6). These results are supportive of the notion that the increase of bone mass in mice with inactivated *Sirt1* in the brain is due to the decrease in SNS signaling in osteoblasts.

### Molecular mechanisms mediating SIRT1 regulation of SNS activity.

**Figure 3:**
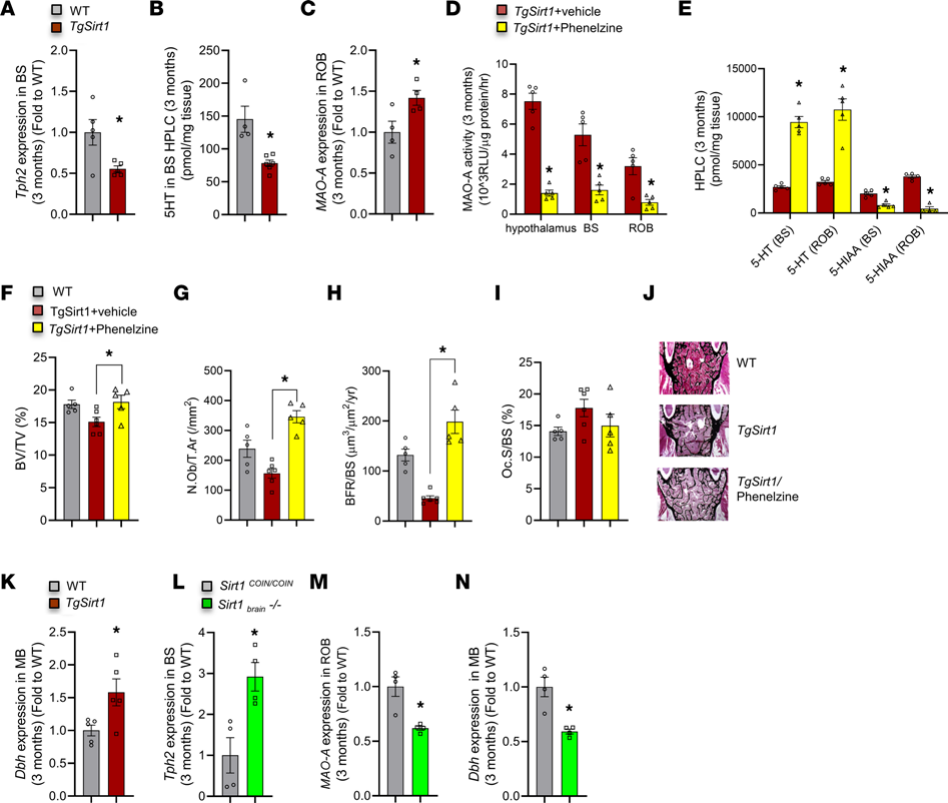
Decreased serotonin synthesis and increased *Dbh* and *MAO-A* expression in the brain of *TgSirt1* mice. (**A**) *Tph2* expression levels in the brain stem (BS) of 3-month-old *TgSirt1* mice (*n* = 5) versus WT controls (*n* = 5). (**B**) 5HT levels in brain stem of *TgSirt1* mice (*n* = 6) versus WT controls (*n* = 4) measured by HPLC. (**C**) *MAO-A* expression levels in the rest of brain (ROB) of 3-month-old *TgSirt1* mice (*n* = 4) versus WT controls (*n* = 4). (**D**) MAO-A activity (10^3^ RLU/μg protein/h) in the hypothalamus, brain stem, and rest of brain of *TgSirt1* mice treated with phenelzine (*n* = 5) versus vehicle controls (*n* = 5). (**E**) 5HT and 5-HIAA levels in brain stem and rest of brain of *TgSirt1* mice treated with phenelzine (*n* = 5) versus vehicle controls (*n* = 5) measured by HPLC. (**F**) BV/TV (%); (**G**) N.Ob/T.Ar (/mm^2^); (**H**) BFR/BS (μm^3^/μm^2^/yr); and (**I**) Oc.S/BS (%) of 3-month-old *TgSirt1* mice treated with phenelzine (*n* = 5) versus vehicle (*n* = 6) and WT controls (*n* = 5). (**J**) Representative images of spines from 3-month-old *TgSirt1* mice treated with phenelzine versus vehicle and WT controls stained with von Kossa. (**K**) *Dbh* expression levels in midbrain (MB) of 3-month-old *TgSirt1* mice (*n* = 5) versus WT controls (*n* = 5). (**L**) *Tph2* expression levels in brain stem of 3-month-old *Sirt1_brain_^–/–^* mice (*n* = 4) versus *Sirt1^COIN/COIN^* controls (*n* = 4). (**M**) *MAO-A* expression levels in rest of brain of 3-month-old *Sirt1_brain_^–/–^* mice (*n* = 4) versus *Sirt1^COIN/COIN^* controls (*n* = 4). (**N**) *Dbh* expression levels in MB of 3-month-old *Sirt1_brain_^–/–^* mice (*n* = 4) versus *Sirt1^COIN/COIN^* controls (*n* = 4). Data are represented as mean ± SEM. (**A**–**E** and **K**–**N**) *P < 0.05, Student’s *t* test. (**F**–**I**) *P < 0.05, *TgSirt1* mice treated with phenelzine versus vehicle by 1-way ANOVA.

To begin deciphering the molecular events triggered by neuronal SIRT1, we examined the involvement of known regulators of SNS activity in its effects in bone. Brain-derived serotonin, whose synthesis depends on the enzyme tryptophan hydroxylase 2 (TPH2) (34), which is present only in brain stem neurons, decreases SNS activity, resulting in increased bone mass. Hence, we determined whether this regulatory loop was affected in *TgSirt1* mice and found that *Tph2* expression was decreased in their brain stems (Figure 3A). Moreover, the brain content of serotonin was decreased in the brain stem of *TgSirt1* mice (Figure 3B). These observations pointed toward a central mode of action of SIRT1 on bone cells possibly mediated through serotonin/SNS signaling.

It has been shown that SIRT1 acts directly in the brain to increase the expression of monoamine oxidase A (*MAO-A*), the major enzyme in the brain that catalyzes degradation of serotonin by converting it to its 5-hydroxyindoleacetic acid (5-HIAA) byproduct (24). Consistent with this observation, expression of *MAO-A* was increased in the brain of *TgSirt1* mice (Figure 3C). A MAO-A contribution to SIRT1 influence on SNS activity and bone mass was tested by treating 3-month-old *TgSirt1* mice with the MAO-A inhibitor phenelzine (35). Phenelzine was administered i.p. (20 mg/kg body weight) every other day for 4 weeks. This regimen suppressed MAO-A activity in the brain and, as a result, it increased serotonin and decreased 5-HIAA levels in *TgSirt1* mice (Figure 3, D and E). The magnitude of these effects was similar throughout different brain areas and to that observed in mice lacking *Sirt1* in the brain (24). In addition, phenelzine reversed the low bone mass in *TgSirt1* mice by restoring both osteoblast and osteoclast numbers to normal levels (Figure 3, F–J). Taken together, these data suggest two nonexclusive mechanisms that would explain the decrease in serotonin content in the brain of *TgSirt1* mice: first, increase of expression of *MAO-A*, a SIRT1 target that catabolizes brain serotonin, and second, downregulation of *Tph2* expression, the rate-limiting enzyme in serotonin synthesis in brain stem raphe nuclei. Thus, SIRT1 acts in the brain stem via *Tph2* or in *MAO-A*–expressing neurons to regulate SNS activity and control bone mass.

In addition to the brain stem, another brain region needed for the regulation of bone mass is the locus coeruleus, in which catecholamine synthesis is initiated by the enzyme dopamine β hydroxylase (DBH), which is highly expressed in neurons of the locus coeruleus, noradrenergic and adrenergic neurons in the brain, the sympathetic ganglia, and adrenomedullary chromaffin cells in peripheral tissues (36). Since *Sirt1* is expressed in the locus coeruleus (Figure 2A), we asked whether at least part of its bone-suppressing actions might be due to increasing *Dbh* expression in this structure. Keeping in mind that *Dbh* mRNA expression may not equate to protein expression, activity, and NE level, we found that *Dbh* expression in the midbrain of *TgSirt1* mice was increased as compared with that in WT littermates (Figure 3K). Moreover, expression of *Tph2*, *MAO-A*, and *Dbh* in mice with adenoviral deletion of *Sirt1* in the brain mirrored our observations in *TgSirt1* mice (Figures 3, L–N).

### Brain stem and MAO-A–expressing neurons, but not locus coeruleus, are the specific brain sites through which SIRT1 regulates skeletal homeostasis throughout aging.

Subsequently, we sought to identify the site of SIRT1 action in the brain through which it influences the sympathetic tone and bone mass. An increase in SNS output could be due to a decrease in brain content of serotonin (Figure 3, A and B) (37) and/or increased *Dbh* expression in the locus coeruleus (Figure 3K). Suppression of serotonin levels may occur through a decrease in the expression of *Tph2* (Figure 3A) (37) and/or an increase in the expression of *MAO-A* (Figure 3C) (24). In identifying the contribution of each pathway to the effect of neuronal SIRT1, we took advantage of the fact that each pathway operates at a distinct site of the brain. Whereas *Tph2* expression and serotonin synthesis occur in the brain stem, *MAO-A* is more widely expressed throughout the brain (24, 38) and affects 5HT levels with a similar magnitude in the brain stem and the rest of the brain (Figure 3E). *Dbh* is expressed in neurons of the locus coeruleus (39–41).

*Sirt1^COIN/COIN^* mice were crossed with *Synapsin-Cre* (42), for inactivation in all neuronal cells; *Sert-Cre* (43), for inactivation specifically in serotonergic neurons, and *Dbh-Cre* (44), for inactivation of Sirt1 in the locus coeruleus. Although *Dbh* is expressed in all catecholamine-producing cells, in the particular *Dbh-Cre* line we used (*Dbh-Cre/9-9*), Cre is solely expressed in the locus coeruleus (44). *Sirt1* was efficiently inactivated in each specific brain locus, as shown by immunofluorescence staining (IFC) analysis of GFP expression and *Sirt1* mRNA expression in each specific appropriate brain area, but not in other tissues (Supplemental Figure 2).

**Figure 4:**
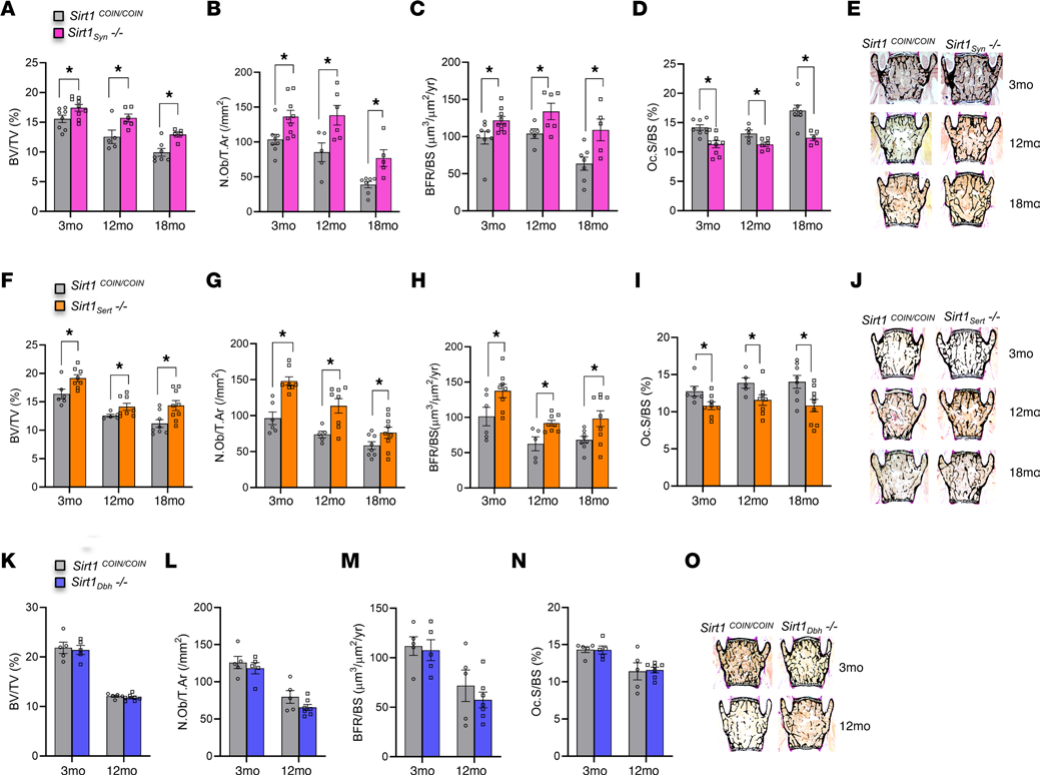
Inactivation of *Sirt1* in serotonergic and *MAO-A*–expressing neurons, but not in the locus coeruleus, increases bone mass in spines of male mice. (**A**) BV/TV (%); (**B**) N.Ob/T.Ar (/mm^2^); (**C**) BFR/BS (μm^3^/μm^2^/yr); and (**D**) Oc.S/BS (%) of 3-, 12-, and 18-month-old male *Sirt1_Syn_^–/–^* mice (3 months: *n* = 9; 12 months: *n* = 6; 18 months: *n* = 5) versus *Sirt1^COIN/COIN^* controls (3 months: *n* = 8; 12 months: *n* = 5; 18 months: *n* = 7). (**E**) Representative images of spines from 3-, 12-, and 18-month-old male *Sirt1_Syn_^–/–^* mice versus *Sirt1^COIN/COIN^* controls, stained with von Kossa. (**F**) BV/TV (%); (**G**) N.Ob/T.Ar (/mm^2^); (**H**) BFR/BS (μm^3^/μm^2^/yr); and (**I**) Oc.S/BS (%) of 3-, 12-, and 18-month-old male *Sirt1_Sert_^–/–^* mice (3 months: *n* = 8; 12 months: *n* = 8; 18 months: *n* = 9) versus *Sirt1^COIN/COIN^* controls (3 months: *n* = 6; 12 months: *n* = 5; 18 months: *n* = 8). (**J**) Representative images of spines from 3-, 12-, and 18-month-old male *Sirt1_Sert_^–/–^* mice versus *Sirt1^COIN/COIN^* controls stained with von Kossa. (**K**) BV/TV (%); (**L**) N.Ob/T.Ar (/mm^2^); (**M**) BFR/BS (μm^3^/μm^2^/yr); and (**N**) Oc.S/BS (%) of 3- and 12- month-old male *Sirt1_Dbh_^–/–^* mice (3 months: *n* = 5; 12 months: *n* = 7) versus *Sirt1^COIN/COIN^* controls (3 months: *n* = 5; 12 months: *n* = 5). (**O**) Representative images of spines from 3- and 12-month-old male *Sirt1_Dbh_^–/–^* mice versus *Sirt1^COIN/COIN^* controls stained with von Kossa. Data are represented as mean ± SEM. *P < 0.05 versus *Sirt1^COIN/COIN^* by Student’s *t* test.

**Figure 5:**
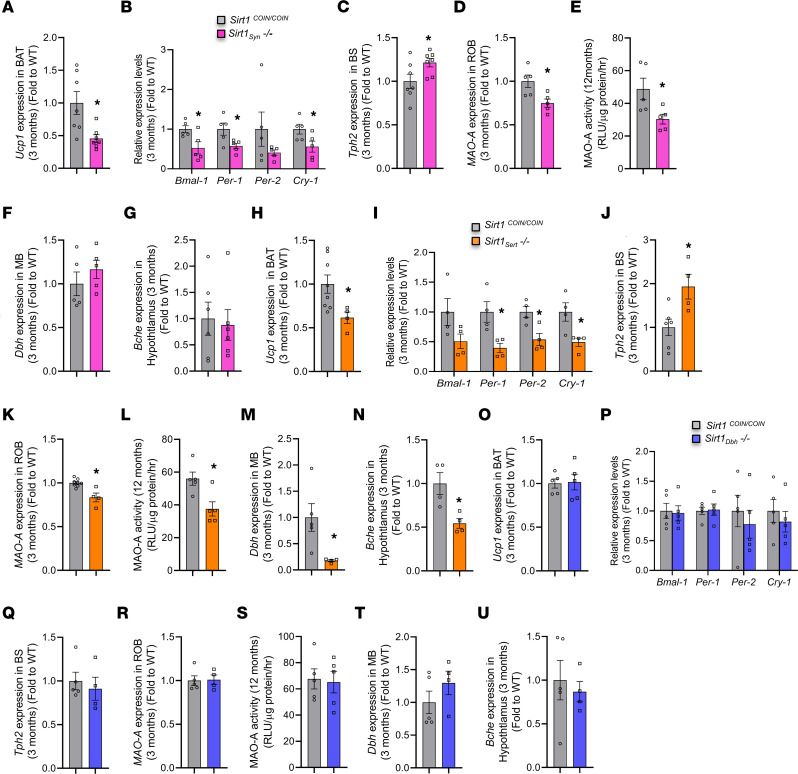
Neuronal SIRT1 decreases bone mass by decreasing serotonin synthesis and enhancing its catabolism through its actions on serotonergic and *MAO-A*–expressing neurons. (**A**) *Ucp1* expression in BAT of *Sirt1_Syn_^–/–^* mice (*n* = 7) versus controls (*n* = 7). (**B**) Expression of SNS target genes in long bone of *Sirt1_Syn_^–/–^* mice (*n* = 5) versus controls (*n* = 5). (**C**) *Tph2* expression in brain stem of *Sirt1_Syn_*^–/–^ mice (*n* = 7) versus controls (*n* = 7). (**D**) *MAO-A* expression and (**E**) MAO-A activity in rest of brain of *Sirt1_Syn_^–/–^* mice (*n* = 5) versus controls (*n* = 5). (**F**) *Dbh* expression in MB of *Sirt1_Syn_^–/–^* mice (*n* = 5) versus controls (*n* = 5). (**G**) *Bche* expression in hypothalamus of *Sirt1_Syn_^–/–^* mice (*n* = 6) versus controls (*n* = 6). (**H**) *Ucp1* expression in BAT of *Sirt1_Sert_^–/–^* mice (*n* = 4) versus controls (*n* = 8). (**I**) Expression of SNS target genes in long bone of *Sirt1_Sert_^–/–^* mice (*n* = 4) versus controls (*n* = 4). (**J**) *Tph2* expression in brain stem of *Sirt1_Sert_^–/–^* mice (*n* = 4) versus controls (*n* = 6). (**K**) *MAO-A* expression in rest of brain of *Sirt1_Sert_^–/–^* mice (*n* = 4) versus controls (*n* = 8). (**L**) MAO-A activity in rest of brain of *Sirt1_Sert_^–/–^* mice (*n* = 5) versus controls (*n* = 5). (**M**) *Dbh* expression in MB of *Sirt1_Sert_^–/–^* mice (*n* = 4) versus controls (*n* = 5). (**N**) *Bche* expression in hypothalamus of *Sirt1_Sert_^–/–^* mice(*n* = 4) versus controls (*n* = 4). (**O**) *Ucp1* expression in BAT of *Sirt1_Dbh_^–/–^* mice (*n* = 5) versus controls (*n* = 5). (**P**) Expression of SNS target genes in long bone of *Sirt1_Dbh_^–/–^* mice (*n* = 5) versus controls (*n* = 5). (**Q**) *Tph2* expression in brain stem of *Sirt1_Dbh_^–/–^* mice (*n* = 4) versus controls (*n* = 5). (**R**) *MAO-A* expression in rest of brain of *Sirt1_Dbh_^–/–^* mice (*n* = 4) versus controls (*n* = 5). (**S**) MAO-A activity in rest of brain of *Sirt1_Dbh_^–/–^* mice (*n* = 5) versus controls (*n* = 5). (**T**) *Dbh* expression in MB and (**U**) *Bche* expression in hypothalamus of *Sirt1_Dbh_^–/–^* (*n* = 4) versus controls (*n* = 5). Data are represented as mean ± SEM. *P < 0.05 versus *Sirt1^COIN/COIN^* by Student’s *t* test.

The skeletal phenotypes of *Sirt1_Syn_^–/–^*, *Sirt1_Sert_^–/–^*, and *Sirt1_Dbh_^–/–^* female and male mice at 3, 12, and 18 months of age were analyzed. Inactivation of *Sirt1* in synapsin- or sert-expressing neurons increased vertebral bone volume by increasing osteoblast numbers and BFR and by suppressing osteoclast numbers (Figure 4, A–J, and Supplemental Figure 3, A–J). The same degrees of bone volume, BFR increase, and bone cell number change were maintained throughout all ages examined (Figure 4, A–J, and Supplemental Figure 3, A–J). Similar changes were observed in the trabecular bone volume analyzed in long bones (proximal tibiae) of 3- and 18-month-old *Sirt1_Syn_^–/–^* and *Sirt1_Sert_^–/–^* mice (Supplemental Figure 4, A and E, and Supplemental Figure 5, A and E) by μCT analysis. In agreement with this, μCT analysis revealed significantly higher trabecular number and lower trabecular separation in tibia trabecular area, while average cortical thickness at the tibia middiaphysis was not altered (Supplemental Figure 4, B–D and F–H, and Supplemental Figure 5, B–D and F–H). In contrast, inactivation of *Sirt1* in the locus coeruleus did not affect trabecular bone mass at the spine or proximal tibia or cortical thickness at tibia middiaphysis at any age (Figure 4, K–O, and Supplemental Figure 3, K–O, Figure 4, I–L, and Figure 5, I–L).

In agreement with these changes in bone mass, SNS activity was decreased in *Sirt1_Syn_^–/–^* and*Sirt1_Sert_^–/–^* mice, as indicated by decreased expression of *Ucp1* in BAT (Figure 5, A and H) and the SNS clock targets in osteoblasts at 3 months of age (Figure 5, B and I). In contrast, *Ucp1* and clock gene expression were not altered in *Sirt1_Dbh_^–/–^* mice (Figure 5, O and P). Moreover, *Tph2* expression in brain stem was increased in *Sirt1_Syn_^–/–^* and *Sirt1_Sert_^–/–^* (Figure 5, C and J) mice, but not altered in *Sirt1_Dbh_^–/–^* mice (Figure 5Q). In parallel, *MAO-A* expression and activity in brain were decreased in both *Sirt1_Syn_^–/–^* and *Sirt1_Sert_^–/–^* mice (Figure 5, D, E, K, and L), but not affected in *Sirt1_Dbh_^–/–^* mice (Figure 5, R and S).

Finally, in agreement with the absence of any changes in bone mass and SNS activity, *Dbh* expression was not affected in midbrain of *Sirt1_Dbh_^–/–^* mice (Figure 5T). In contrast, *Dbh* expression was decreased in midbrain of *Sirt1_Sert_^–/–^* mice (Figure 5M), suggesting that SIRT1 indirectly regulates *Dbh* expression by increasing serotonin levels. Moreover, hypothalamic expression of butyrylcholinesterase (*Bche*), a gene downregulated by brain serotonin signaling (45, 46), was decreased in *Sirt1_Sert_^–/–^* mice but not in *Sirt1_Dbh_^–/–^* mice (Figure 5, N and U). Expression of *Dbh* and *Bche* was not altered in *Sirt1_Syn_^–/–^* mice (Figure 5, F and G).

These results show that catecholamine synthesis in locus coeruleus is not a target of neuronal SIRT1, and therefore, inactivation of *Sirt1* in this brain region does not affect bone mass. Instead, inactivation of *Sirt1* in serotonergic or *MAO-A*–expressing neurons increases neuronal serotonin levels in brain by increasing its synthesis through increased expression of *Tph2* or by decreasing its catabolism through increased expression of *MAO-A*. Increased levels of serotonin suppress SNS activity, leading to increased bone mass caused by increasing bone formation and suppressing bone resorption.

### Central effects of SIRT1 are dominant over its peripheral direct effects in bone cells on the regulation of bone mass in the aging skeleton.

**Figure 6:**
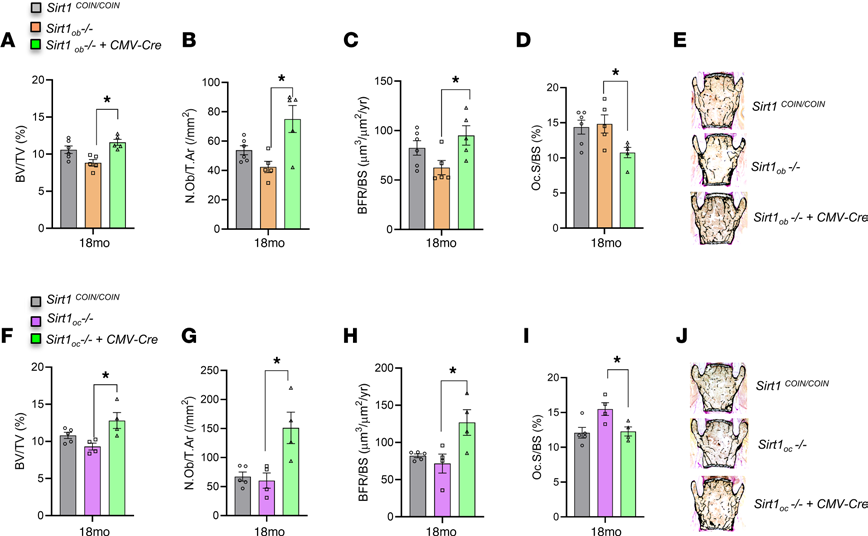
Central effects of SIRT1 are dominant over its peripheral direct effects in bone cells on the regulation of bone mass in the aging skeleton. (**A**) BV/TV (%); (**B**) N.Ob/T.Ar (/mm^2^); (**C**) BFR/BS (μm^3^/μm^2^/yr); and (**D**) Oc.S/BS (%) of 18-month-old Adeno-CMV-Cre i.c.v. injected *Sirt1_ob_^–/–^* mice (*n* = 5) versus vehicle (*n* = 5) and *Sirt1^COIN/COIN^* controls (*n* = 6). (**E**) Representative images of spines from 18-month-old Adeno-CMV-Cre i.c.v. injected *Sirt1_ob_^–/–^* mice versus vehicle and *Sirt1^COIN/COIN^* controls stained with von Kossa. (**F**) BV/TV (%); (**G**) N.Ob/T.Ar (/mm^2^); (**H**) BFR/BS (μm^3^/μm^2^/yr); and (**I**) Oc.S/BS (%) of 18-month-old Adeno-CMV-Cre i.c.v. injected *Sirt1_oc_^–/–^* mice (*n* = 4) versus vehicle (*n* = 4) and *Sirt1^COIN/COIN^* controls(*n* = 5). (**J**) Representative images of spines from 18-month-old Adeno-CMV-Cre i.c.v. injected *Sirt1_oc_^–/–^* mice versus vehicle and *Sirt1^COIN/COIN^* controls stained with von Kossa. Data are represented as mean ± SEM. *P < 0.05, Adeno-CMV-Cre i.c.v. injected *Sirt1_ob_^–/–^* or *Sirt1_oc_^–/–^* mice versus vehicle by 1-way ANOVA.

Studies in *Sirt1*-deficient mice suggested that SIRT1 promotes osteoblast and suppresses osteoclast numbers by direct actions on these cells (20–22). In parallel, we found that SIRT1 decreases levels of brain serotonin, which promotes bone mass by suppressing sympathetic tone. Therefore, we sought to determine genetically the contribution of SIRT1 direct effects in bone cells versus its neuronal effects on the aging skeleton. For this purpose, we inactivated *Sirt1* in osteoblasts (*Sirt1_Ob_^–/–^* mice) or osteoclasts (*Sirt1_Oc_^–/–^* mice) using *Col1a1-Cre* and *CD11b-Cre* mice, respectively (47, 48). *Sirt1* was inactivated in the brain of 17-month-old *Sirt1_Ob_^–/–^* or *Sirt1_Oc_^–/–^* mice by injection of *Cre*-expressing adenovirus to the third ventricle (anterior-posterior, –1.82 mm; lateral, 0.00 mm; and ventral, –5.35 mm relative to bregma, ref. 49). Mice were harvested 4 weeks after injection. Assessment of bone mass in vehicle-injected 18-month-old *Sirt1_Ob_^–/–^* and *Sirt1_Oc_^–/–^* mice showed that inactivation of *Sirt1* in osteoblasts decreased bone formation and osteoblast numbers (Figure 6, A–E) and its inactivation in osteoclasts increased osteoclast numbers (Figure 6, F–J). However, inactivation of *Sirt1* in the brain of either *Sirt1_Ob_^–/–^* or *Sirt1_Oc_^–/–^* mice overruled the effects of peripheral deletion in either cell type, reversed the bone phenotype, and led to an increase in bone mass that was due to an increase in osteoblast numbers and BFR and a decrease in osteoclast numbers (Figure 6, A–J). These results indicate that neuronal SIRT1 overrides the actions of bone cell–expressed *Sirt1* *i*n aged mice.

## Discussion

Previous studies have shown that SIRT1 regulates bone remodeling by acting directly on osteoblasts and osteoclasts (20–22, 50). Our studies support these conclusions, showing that osteoblast- or osteoclast-specific deletion of *Sirt1* decreases bone formation and osteoblast numbers or increases osteoclast numbers, respectively. However, our observations broaden the importance of SIRT1-dependent regulation of bone mass by showing that it acts through a second and unanticipated mechanism. They demonstrate that SIRT1 indirectly affects bone cell function by regulating SNS tone. The increase in SNS output is due to a decrease in content of brain serotonin, a neurotransmitter that promotes bone mass by suppressing sympathetic tone (37). Neuronal SIRT1 regulates serotonin levels by suppressing expression of *Tph2*, the rate-limiting enzyme in brain serotonin synthesis, and by upregulating *MAO-A* expression and thus promoting serotonin catabolism (24). That SIRT1 controls bone mass by opposite actions in osteoblasts and osteoclasts versus the SNS suggests that SIRT1 finely tunes a balance in skeletal actions, perhaps by responding to different environmental stimuli, depending on its site of expression.

These conclusions are derived from our observations showing that both TPH2 and MAO-A mediate the suppressing actions of neuronal SIRT1 in brain serotonin levels. SIRT1 acts in the brain stem to reduce the expression of *Tph2*, as *Tph2* expression is decreased in the brain stem of *TgSirt1* mice and increased in the brain stem of *Sirt1_Sert_^–/–^* mice, and following adenoviral inactivation of *Sirt1* in the brain. This could be due to a direct effect of SIRT1 through deactylation of factors involved in *Thp2* transcriptional regulation and/or indirect effects, such as regulation of circadian rhythm or stress hormones and glucocorticoids by SIRT1 that are known to regulate *Tph2* expression (51–58).

In addition, SIRT1 also acts through MAO-A to promote serotonin catabolism, as shown by the following: *MAO-A* expression and activity are increased in the brain of *TgSirt1* mice and decreased in the brain of *Sirt1_Sert_^–/–^* mice and following adenoviral inactivation of *Sirt1* in the brain; and pharmacological inhibition of MAO-A activity by phenelzine in *TgSirt1* mice rescues their low bone mass by increasing osteoblast numbers and decreasing osteoclast numbers to levels equal to those of WT mice. SIRT1 has been shown to increase *Mao-a* gene expression in the brain by deacetylating and thus activating the transcription factor NHLH2, which subsequently binds on the *Mao-a* promoter to upregulate *Mao-a* expression (24). No obvious behavioral abnormalities were observed that could have an indirect impact on bone mass. Moreover, catecholamine synthesis in the locus coeruleus is not a target of neuronal SIRT1, and therefore, inactivation of *Sirt1* in this brain region does not affect bone mass.

Previous studies have shown that SIRT1 promotes bone formation and decreases bone resorption, acting in a cell-autonomous manner in both osteoblasts and osteoclasts. These effects were attributed to the ability of SIRT1 to suppress NF-κB signaling in both osteoblasts and osteoclasts (22) and enhance FoXO1-mediated transcription in osteoclasts (50). Enhancement of FOXO3A-mediated *Runx2* gene transcription by SIRT1 has also been suggested as promoting osteoblast differentiation of mesenchymal stem cells (MSCs) (20). Moreover, global *Sirt1* haploinsufficiency was reported to decrease bone formation in female mice only, due to reduced osteoblast activity attributed to suppression of expression of sclerostin (*Sost*), an inhibitor of bone formation by SIRT1 (21). No effects, though, were observed in male mice, despite a similar alteration in *Sost* expression, suggesting a role for sex steroids and additional factors in these effects. A role for estrogen in the regulation of *Sost* has been described and vice versa (59, 60). The absence of a bone phenotype in male mice also suggests that *Sirt1* expression in additional tissues may affect skeletal development and counteract the effects of osteoblast- and osteoclast-specific SIRT1. Indeed, our studies support these conclusions showing that osteoblast- or osteoclast-specific deletion of *Sirt1* using the collagen-type 1 2.3 kb promoter or the Cd11b promoter, respectively, decreased bone formation and osteoblast numbers and increased osteoclast numbers. However, they broaden the importance of SIRT1-dependent regulation of bone mass by showing that it acts through the brain to regulate bone remodelling.

Notably, our experiments inactivating *Sirt1* in aged mice with osteoblast- or osteoclast- specific deletion of *Sirt1* show that neuronal SIRT1 has a dominant effect over peripheral, bone-expressed protein in the regulation of bone mass. This observation agrees with known instances in which a molecule regulates bone mass through both central and peripheral actions; it is the central action that overrides the peripheral one. This is exemplified by the following: leptin, since leptin deficiency suffices to overcome, in mice and humans, the deleterious effects of gonadal insufficiency on bone mass accrual (3–6); adiponectin, in which case peripheral actions of adiponectin manifest up until 3 months of age, whereas central actions of the hormone become dominant after 6 months of age (61); and orexin, which regulates bone remodeling via a dominant positive central action that overarches the negative peripheral one (62). In addition, *Sirt1* expression in bone decreases with aging in mice (22), which, along with the parallel increase in SNS outflow with aging (12–14), suggests an overall adverse effect on bone mass conferred by the central actions of Sirt1.

Upregulation of SNS signaling has detrimental effects on bone mass and strength, and conversely, patients on beta blockers have increased BMD and reduced fracture risk (7–10). These studies may suggest that modulating sympathetic signaling in bone cells can influence bone mass accrual and maintenance in humans, as it does in mice. At this time, this concept has not been decisively demonstrated and studies indicating a beneficial clinical outcome are still needed. A first interventional study (11) suggested beneficial effect on BMD and bone microarchitecture in patients treated clinically with adrenergic receptor–selective blockers compared with nonusers and relative to those receiving placebo. Changes in lumbar spine or femur BMD were not significant, perhaps due to the relatively small number of subjects (~30 per group) and short duration of the study. Based on these findings, a large, nationwide, NIH-funded clinical trial is currently ongoing (Atenolol for the Prevention of Osteoporosis [APO], ClinicalTrials.gov NCT04905277; ref. 63). If results of this prospective study confirm and extend those observed in the pilot study, then beta blockers could fill a crucial clinical need in the primary prevention of osteoporosis. In this case, by showing that brain-expressed *Sirt1* modulates SNS output, our studies identify another aspect of regulation of the SNS effect on bone remodeling and hence may point to other therapeutic strategies for modulating its negative influence on bone mass. These findings would be especially relevant to age-related bone loss, as sympathetic output increases during aging (12–14).

A beneficial effect of resveratrol, a SIRT1 activator, on BMD has been reported (64–66). Multiple biological effects and mechanisms of action for resveratrol have been reported, including activation of estrogen receptor signaling (67, 68), PARP1 and tyrosyl-tRNA synthetase (69), AMPK (70), and MAPK signaling (71). This could be due to the higher bioavailability of SIRT1 in the periphery as compared with the brain, favoring its local direct effects on bone, but also due to additional, SIRT1-independent actions (72–74).

A link between SIRT1 functions in brain and the regulation of bone remodeling would also find support in another poorly understood clinical observation. Cross-sectional and prospective data in large cohorts of men and women taking selective serotonin reuptake inhibitors (SSRIs) have shown that bone density is reduced and fracture risk is increased in these subjects as compared with nonusers or users of other classes of antidepressants (75–79). Although the study of SSRI signaling and influence on bone mass is confounded by the depressive state of these patients, the differential potency of different SSRIs in males versus females, and at distinct skeletal sites, the general outcome of their action is an increase in fracture risk (79). Given that SSRIs act by enhancing serotonin signaling in the brain, these observations appear to contradict the beneficial effect on bone mass accrual associated with brain serotonin (37). On the contrary, as shown recently, because HTR2C, the serotonin receptor mediating the specific effects of brain serotonin on bone (80, 81), becomes desensitized following SSRI treatment, their effect leads to a decrease of serotonin signaling in the brain-bone axis (45). In turn, this deficit causes an increase in sympathetic output and thereby bone loss. These observations further support the contention that upregulation of SNS signaling is detrimental for the skeleton. They also render our studies physiologically and clinically relevant, as they reveal that *Sirt1* activation in the brain mirrors the effects of SSRIs on the skeleton. The brain-specific *Sirt1*-deficient mice can further be used as a model to dissect the basis of sex-, site-, and age-specific effects of SSRIs.

Our observations have several implications. First, they identify a pathway by which different organs interact to control bone mass. Because SIRT1 senses a plethora of environmental signals, this pathway is likely to find applications in aging-associated loss of bone mass, a multifactorial process, and in several bone diseases. Second, our studies identify a mechanism of SIRT1 action that extends beyond its redox-regulating, prosurvival, and metabolic-sensing properties, to mediate organ communications. Finally, the identification of SIRT1 as a regulator of SNS activity can have implications in both the bone and energy metabolism fields. This becomes more important in view of the fact that there are not many endogenous regulators of sympathetic tone that can be manipulated to control its activity. Yet plasma NE levels marked rise with age, suggesting that increased sympathetic signaling in osteoblasts contributes to age-related changes in bone remodeling. Small molecules have been identified that specifically and selectively inhibit or activate SIRT1 activity in vivo (15, 82). Therefore, results from our studies could have important therapeutic applications in the understanding of skeletal changes associated with SSRIs and in the treatment of skeletal and metabolic diseases, the incidence of which steadily increases with aging.

## Methods

### Mice.

*TgSirt1* mice have been previously described (26). A conditional null allele of *Sirt1* with EGFP as a post-Cre reporter was generated using the COIN method (31–33) (Supplemental Figure 1A). The COIN module comprises the 3′ splice region of the rabbit β-globin gene (HBB_RABIT), followed by T2A-*EGFP* and the polyadenylation region (pA) from HBB_RABIT, all placed in the antisense strand of an artificial intron derived from HBB_RABIT. *Lox71* (L71) is placed upstream of the pA, whereas *Lox66* (L66) is placed in a nonconserved region within intron 4 of *Sirt1* and in a manner such that *Lox71* and *Lox66* are in a mirror-image orientation, enabling inversion by Cre. The COIN allele of *Sirt1* was engineered by inserting the COIN module intron into the third protein-coding exon of *Sirt1* (isoform 002, ENSMUST00000105442). Exon 3 carries the catalytic domain of *Sirt1*, and its deletion effectively silences *Sirt1* expression (24). More precisely, the COIN module intron was inserted between coordinates 62798484 and 62798483, thereby splitting exon 3 (ENSMUSE00000453126) into 2 exons, 3L and 3R of 141 and 234 bp, respectively. For BHR and targeting, an FRT-flanked *neo* cassette has been incorporated into the COIN module intron. *Neo* is removed in mice by breeding to an FLPe deleter to give rise to the *Sirt1^COIN^* allele. As the COIN module is antisense to *Sirt1*, it does not interfere with expression of *Sirt1*. However, when inverted by Cre into the sense orientation, it acts as a transcriptional block and at the same time allows the expression of an adjacent EGFP. Briefly, the BAC-based targeting vector was assembled on BAC bMQ-285g15, which encompasses *Sirt1*. The resulting modified BAC was used to generate a conventional targeting vector by subcloning an approximately 15 kb fragment into pMC1-DTA (gift of Yuji Mishina, University of Michigan, Ann Arbor, Michigan, USA). This targeting vector was electroporated into CSL3 (129S6) ES cells, which were then screened for targeting by Southern blot analysis (Supplemental Figure 1B). Targeted *Sirt1^COINneo/+^* ES cells were used to generate chimeras, which were subsequently bred to an FLPe deleter (83) to generate *Sirt1^COIN^* allele–bearing mice. This allele was passed into the germline by further breeding to C57BL/6. *Sirt1^COIN/COIN^* mice are born in Mendelian ratios and have no obvious phenotypes or any discernible physiologic differences from their WT littermates.

*Sirt1^COIN/COIN^* mice were crossed with *Synapsin-Cre* (42), for inactivation in all neuronal cells; *Sert-Cre* (43), for inactivation specifically in serotonergic neurons; *Dbh-Cre* (44), for inactivation of Sirt1 in the locus coeruleus; *Col1a1-Cre* (47), for inactivation specifically in osteoblasts; and *Cd11b-Cre* (48), for inactivation specifically in osteoclasts. *Sirt1* heterozygous mice were intercrossed and animals homozygous for Sirt1 deletion in MAO-A expression neurons (*Sirt1_Syn_^–/–^*), serotonergic neurons (*Sirt1_Sert_^–/–^*), locus coeruleus (*Sirt1_Dbh_^–/–^*), osteoblasts (*Sirt1_Ob_^–/–^*), and osteoclasts (*Sirt1_Oc_^–/–^*) were obtained. *Sirt1_brain_^–/–^* mice were achieved by injection of Adeno-CMV-Cre virus into the third ventricle of 3-month-old *Sirt1^COIN/COIN^* mice. *Sirt1* inactivation in the brain of 18-month-old *Sirt1_Ob_^–/–^* and *Sirt1_Oc_^–/–^* mice was achieved by injection of Adeno-CMV-Cre virus into the third ventricle of the 17-month-old mice. *Dbh-Cre* mice (RBRC01492; Riken) were obtained from the Japan Riken RBC Experimental Animal Division, RIKEN BioResource Center. *Sert-Cre* mice (014554, B6.129[Cg]-Slc6a4<tm1[cre]Xz>/J, JAX) and *Synapsin-Cre* mice (003966, B6.Cg-Tg[Syn1-cre]671Jxm/J, JAX) were obtained from Jackson Laboratories. *CD11b-Cre* mice were obtained from Jean Vacher (Montreal Clinical Research Institute [IRCM], Montreal, Canada). All mice were housed under standard laboratory conditions (12-hours light/12-hours dark; light on at 7:00 am) and temperature-controlled environment with food and water available ad libitum. For each experiment, the mice used were of the same genetic background, as they were all littermates. Mouse genotypes were determined by PCR; primer sequences are listed in Supplemental Table 1.

### Brain injections.

Three-month-old *Sirt1^COIN/COIN^* mice were injected with Adeno-CMV-Cre virus (10^8^ PFU/ml/5 min) using a stereotaxic table. Mice were anesthetized by i.p. injection of ketamine/xylazine (100 mg per kg/10 mg per kg body weight) and placed on a stereotaxic instrument (Stoelting). The calvaria was exposed, a 0.7 mm hole was drilled, and a fine glass pipette containing a high titer (1 × 10^8^ infectious units per ml) of Adeno-CMV-Cre virus in buffer connected to a syringe was driven through the hole in the skull using the stereotactic manipulator. A volume of 1 μl of the viral mixture was injected stereotaxically into the third ventricle with the following coordinates relative to bregma: anterior-posterior, –1.82 mm; lateral, 0.00 mm; and ventral, –5.35 mm. After injection, the pipette was removed and the scalp was sutured.

### Propranolol and phenelzine treatment.

Propranolol (P0884-1G) and phenelzine (P6777-5G) for the treatment of 3-month-old *TgSirt1* mice and WT littermates were purchased from Sigma-Aldrich. Propranolol was administered s.c. at a dose of 5 mg/kg body weight every day for 4 weeks. Phenelzine was administered i.p. at a dose of 20 mg/kg body weight every other day for 4 weeks.

### Measurement of biomarkers.

Mouse urine samples were collected in the morning for at least 3 days within the week prior to sacrifice. Urine epinephrine and NE levels were measured with the Bi-CAT ELISA Kit (Alpco, 17-BCTHU-E02.1) and were normalized with a concentration of creatinine quantified with the Creatinine EIA Kit (Quidel, 8009).

### HPLC chromatography.

5-HT and 5-HIAA were measured in brain stem sections and sections from the rest of the brain using reverse-phase HPLC with electrochemical detection, as previously described (84). The samples were homogenized in 0.5 ml ice-cold 0.4M perchloric acid and centrifuged (5 minutes at 14,000*g*). A 50 μl aliquot of the supernatant was injected over a Waters HPLC system. The mobile phase contained 0.75 mM sodium phosphate (pH 3.1), 1.4 mM 1-octanesulphonic acid, 10M ethylenediaminetetraacetic acid (EDTA), and 8% acetonitrile. The flow rate was 1.0 ml/min. A standard curve was generated with external standards. Values were calculated based on peak area and compared with the standard calibration. The inter- and intraassay coefficients of variation of the assay were less than 5%. The sensitivity was less than 0.5 pmol.

### Measurement of brain MAO-A activity.

For the measurement of MAO-A activity, 5 μg of mitochondrial protein sample was assayed in 96-well plates using the MAO-Glo Kit (Promega, V1401) according to the manufacturer’s instructions and as previously described (85). Briefly, mouse brains were taken and frozen in liquid nitrogen. Hypothalamus, brain stem, and the rest of the brain were dissected, and mitochondrial proteins were isolated by sequential centrifugation for 10 minutes at 3,000 *g* at 4°C to pellet nuclei and any unbroken cells, followed by centrifugation of the supernatant for 15 minutes at 10,000 *g* at 4°C. The resulting pellet was washed in lysis buffer (70 mM sucrose, 230 mM mannitol, 1 mM EDTA pH 7.0, 10 mM Tris-HCl pH 7.5, 1× protease inhibitors; Roche) and centrifuged for another 15 minutes at 10,000 *g* at 4°C. Mitochondrial proteins were incubated with substrate for 1 hour at room temperature. The reaction was stopped by addition of reconstituted luciferin detection reagent and incubation for 20 minutes at room temperature. The amount of light produced was recorded by using a microplate luminometer, and MAO-A activity in RLU/μg protein/h was calculated.

### Histological analysis: bone histomorphometry analysis and brain sample imaging.

Bone histomorphometry analysis was performed as previously described (86). Calcein (MilliporeSigma, C0875-5G) was dissolved in 0.15M NaCl, 2% NaHCO_3_ and was injected i.p. at 0.025 mg/g body weight on day 1 and day 4. Then mice were sacrificed on day 6. For bone histomorphometry analysis, L3–L4 vertebrae were fixed for 24 hours in 10% formalin, dehydrated in a graded series of ethanol, and embedded in methyl methacrylate resin. Von Kossa/Van Gieson staining was performed on 7 μm bone sections. Photographs were taken using a Leica DM4000B microscope. Bone volume over total volume (BV/TV) was quantified using ImageJ software (NIH). Three 5 μm bone sections from each mouse were stained with toluidine blue or tartrate-resistant acid phosphatase (TRAP) or received no staining (calcein labeling) for analysis of the parameters of osteoblasts, osteoclasts, and BFR, respectively. Quantification of all 3 parameters was performed with Osteomeasure software (XP v 1.2.0.3.) from Osteometrics, and a Leica DMLB microscope outfitted with Sony DXC390 color video camera was used.

For cryosection preparation, brains isolated from mice were perfused with 4% PFA/PBS, fixed in 4% PFA/PBS for 16 hours at 4°C, cryoprotected in 30% sucrose/PBS overnight, embedded in cryomatrix (Fisher Scientific, 4585), and sectioned at 30 μm. Sections were stained following standard immunofluorescence protocol. Antibodies used were as follows: anti-GFP mouse monoclonal antibody (Takara, catalog 632375); anti-Sirt1 rabbit antibody (Cell Signaling Technology, catalog 2028S); anti-Tph2 rabbit polyclonal antibody (Novus NB, catalog 100-74555); Alexa Fluor 488 AffiniPure donkey anti-mouse IgG (Jackson ImmunoResearch, catalog 715-545-150); and Alexa Fluor 594 AffiniPure donkey, anti-rabbit IgG (Jackson ImmunoResearch, catalog 711-585-152). For all antibodies, validation is provided at the manufacturer’s website.

### Tibia μCT analysis.

Tibia trabecular and cortical bone architecture were assessed by μCT (vivaCT 80, SCANCO Medical AG) using energy settings of 55 kVp, 145 μA, a 0.5 mm Al filter, and an integration time of 250 ms. Images were reconstructed at an isotropic voxel size of 10.4 μm. The trabecular bone volume of interest (VOI) was 145 μCT slices, corresponding to a 1.5 mm region in the longitudinal direction, starting below the growth plate of the proximal tibia. The cortical bone VOI was 97 μCT slices, corresponding to a 1.0 mm region in the longitudinal direction, centered at the tibia middiaphysis. The total tibia lengths were measured from the μCT scout view. Grayscale images were subjected to a Gaussian filter (sigma = 0.8, support = 1) to reduce noise, and a global threshold was applied to binarize grayscale images (36% and 40% maximum grayscale value for trabecular and cortical bone, respectively). The trabecular and cortical VOIs were evaluated by 3D standard microstructural analysis using the manufacturer’s software.

### Gene expression analysis.

RNA isolation, cDNA preparation, and real-time PCR analyses were carried out following standard protocols. For bone tissue analysis, bone marrow cells were removed completely by extensively flushing the femurs with PBS. TRIzol reagent was used for RNA isolation, the Random Hexamers cDNA Synthesis Kit (Takara, 639546) was used for reverse-transcription PCR, and PowerUp SYBR Green Master Mix (Applied Biosystems, 100029284) was used for quantitative PCR. β-Actin was used as an internal control. Data are presented as fold change over control, unless otherwise indicated. Melting curves analysis was performed in every experiment for verification of amplicon specificity. Primer sequences are listed in Supplemental Table 2.

### Statistics.

Results are presented as mean ± SEM. Group sizes were determined by performing a power calculation to lead to an 80% chance of detecting a significant difference (*P* ≤ 0.05). A *n* = 3 was used in the 1.5-month-old group of WT mice that were used as controls for the same age TgSirt1 mice. The WT values are within the same range we have always observed for WT mice at this age. Moreover, the difference we observed at 1.5 months of age among WT and TgSirt1 mice was consistent with and preserved at 3 and 12 months of age. Unpaired, 2-tailed Student’s *t* test was performed for comparisons between 2 groups and 1-way ANOVA for comparisons of more than 2 groups. For all experiments, *P* ≤ 0.05. Analyses were performed using GraphPad Prism for ANOVA and Excel (Microsoft) for Student’s *t* test.

### Study approval.

All animal procedures were approved by the Columbia University Animal Care and Use Committee.

## Author contributions

NL and SK initiated the study, designed experiments, and analyzed data. NL and IM performed experiments and analyzed data. MC performed bone phenotype analysis of phenelzine-treated TgSirt1 mice. PTS performed the tibia μCT analysis under XEG’s supervision. BB performed some qPCR experiments. CSL and AE generated the Sirt1^COIN/COIN^ mice. YYH and JJM performed brain HPLC and MAO activity. IM, NL, and SK wrote the manuscript. SK directed the research.

## Supplementary Material

Supplemental data

## Version 1 10/04/2022

In-Press Preview

## Version 2 12/01/2022

Electronic publication
