# Supplementary material for: A neuronal action of sirtuin 1 suppresses bone mass in young and aging mice
Source: J Clin Invest. 2022 Dec 1;132(23):e152868. doi: 10.1172/JCI152868 (PMC9711874; doi:10.1172/JCI152868)
Supplement: Supplemental data [file jci-132-152868-s155.pdf]

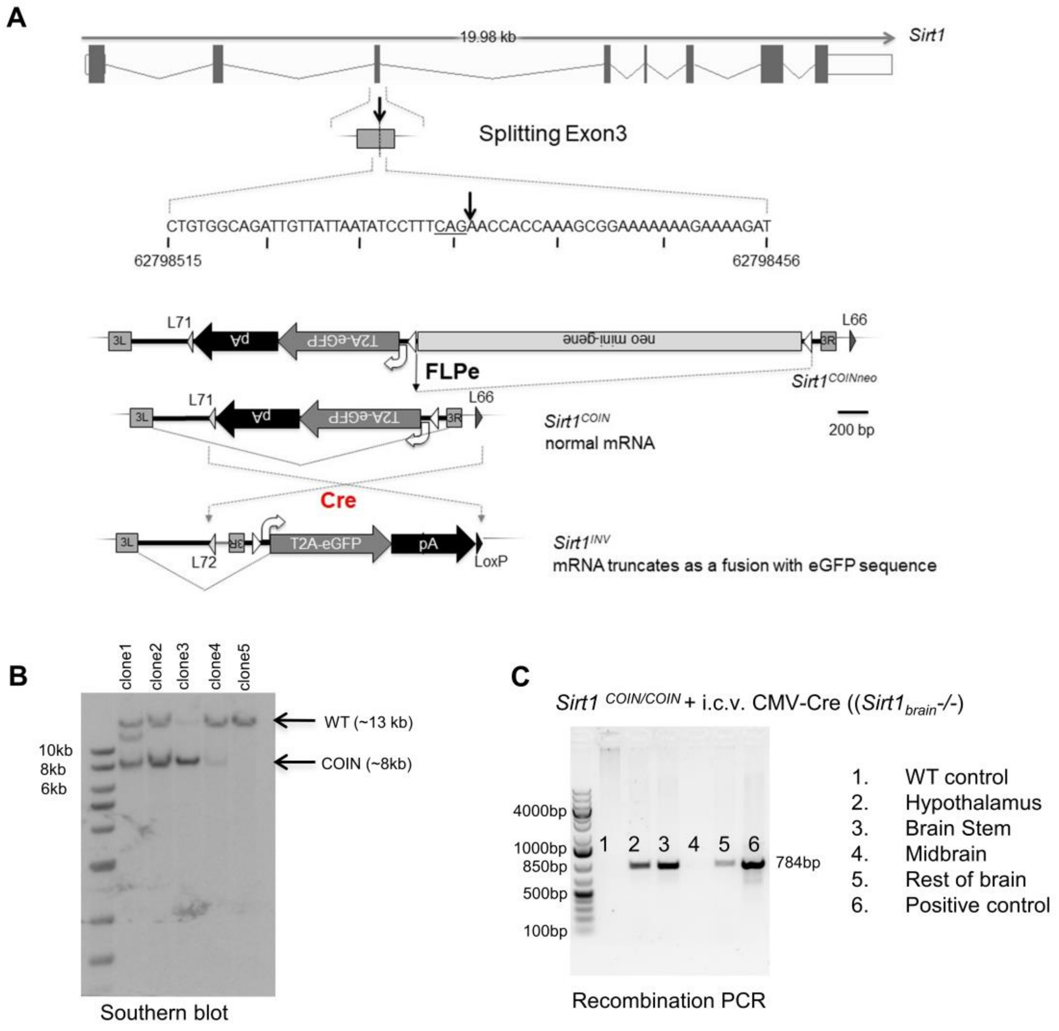

Supplemental Figure 1. Targeting strategy used to generate a conditional-null allele of *Sirt1* and efficiency of adenovirus-mediated deletion of *Sirt1* in the brain (A) A conditional-null allele of *Sirt1* with eGFP as a post-Cre reporter was generated using the method of Conditional by Inversion (COIN). The COIN module within an artificial intron was placed in the antisense strand of *Sirt1* in exon 3, thereby splitting exon 3 into two operational halves. When placed in the antisense orientation, the COIN module is stealth to transcription. However, when inverted by Cre into the sense orientation, it acts as a transcriptional block and at the same time allows the expression of an adjacent eGFP. (B) Detection of the *Sirt1* COIN allele by Southern blot analysis of genomic DNA extracted from targeted ES cell clones. (C) Detection of the recombined allele by PCR analysis of genomic DNA extracted from hypothalamus, brainstem, midbrain and rest of brain of Adeno-CMV-Cre i.c.v. injected $Sirt1^{COIN/COIN}$ ($Sirt1_{brain}$-/-) mice.

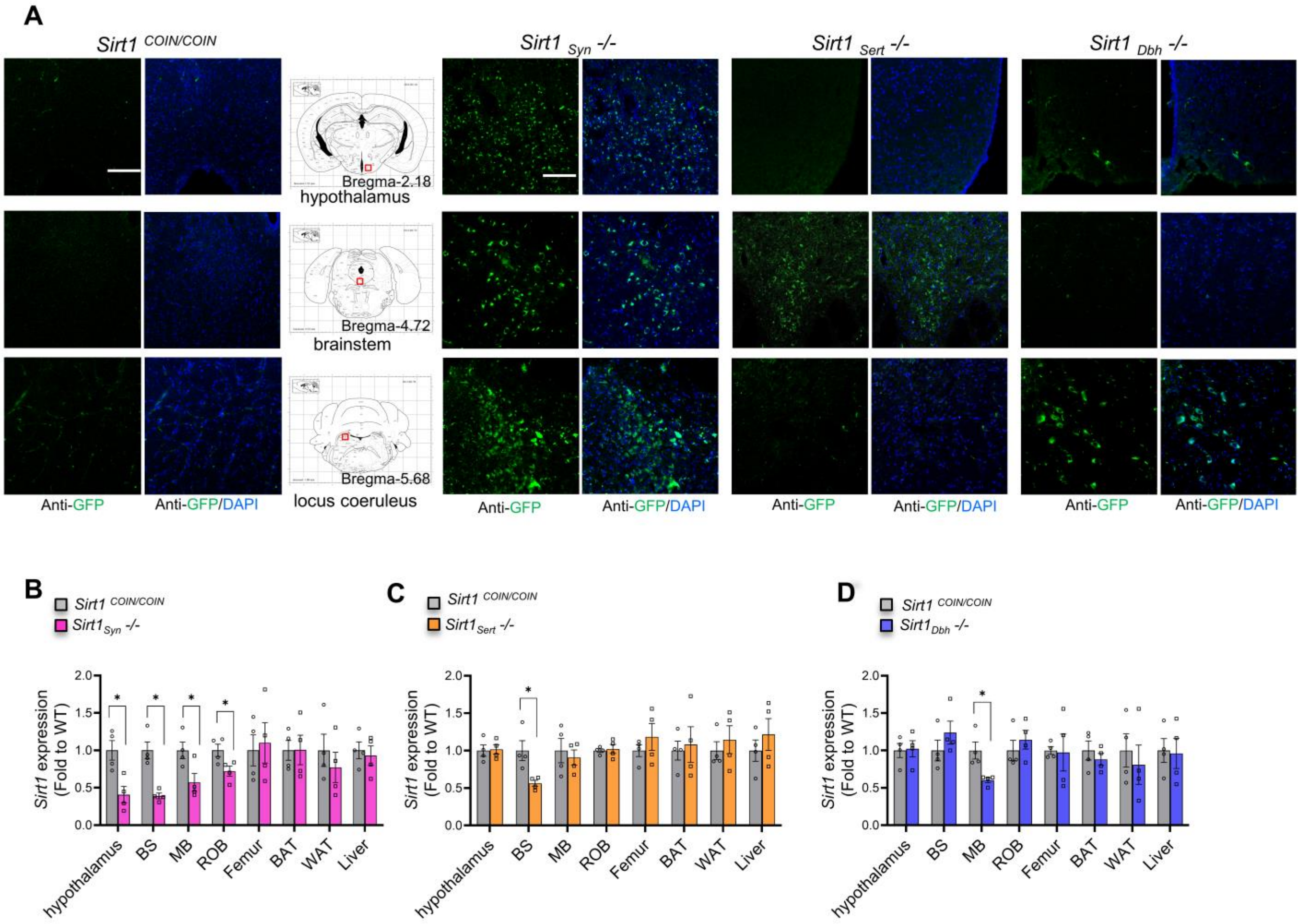

Supplemental Figure 2. Specific inactivation of *Sirt1* in *$Sirt1_{Syn}$ -/-, $Sirt1_{Sert}$ -/- and $Sirt1_{Dbh}$-/-* mice. (A) GFP immunostaining of brain sections of *$Sirt1_{Syn}$ -/-, $Sirt1_{Sert}$ -/-* and *$Sirt1_{Dbh}$ -/-* mice vs *$Sirt1^{COIN/COIN}$* controls**.** The same images of *$Sirt1^{COIN/COIN}$* controls are used in Figure 2B. Scale bar: 100μm. (B) Relative expression of *Sirt1* in hypothalamus, BS, MB, ROB, Femur, BAT, WAT (White Adipose Tissue) and liver of 3-month-old *$Sirt1_{Syn}$ -/-* mice (n=4) vs *$Sirt1^{COIN/COIN}$* controls (n=4)**.** (C) Relative expression of *Sirt1* in hypothalamus, BS, MB, ROB, Femur, BAT, WAT and liver of 3-month-old *$Sirt1_{Sert}$ -/-* mice (n=4) vs *$Sirt1^{COIN/COIN}$* controls (n=4)**.** (D) Relative expression of *Sirt1* in in hypothalamus, BS, MB, ROB, Femur, BAT, WAT and liver of 3-month-old *$Sirt1_{Dbh}$ -/-* mice (n=4) vs *$Sirt1^{COIN/COIN}$* controls (n=4)**.**
Data are presented as mean ± SEM. *: p<0.05 vs *$Sirt1^{COIN/COIN}$* by Student's *t* test.

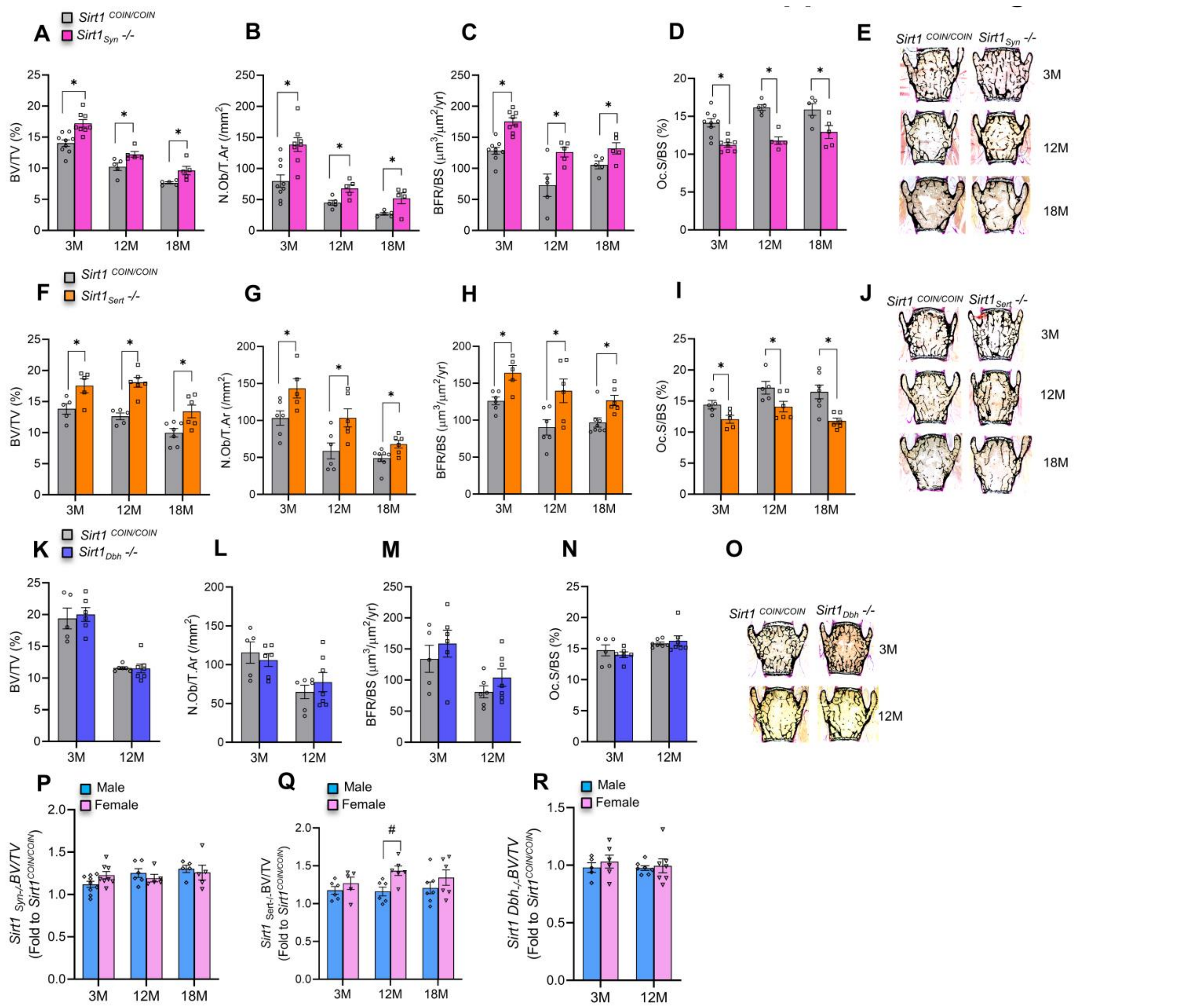

Supplemental Figure 3. Inactivation of *Sirt1* in serotonergic and *MAO-A*-expressing neurons, but not in the locus coeruleus, increases bone mass in spines of female mice. (A) BV/TV (%); (B) N.Ob/T.Ar (/mm^2) ; (C) BFR/BS (µm^3/µm^2/yr) and (D) Oc.S/BS (%) of 3-, 12- and 18-month-old female $Sirt1_{Syn}$ -/- mice (3M: n=8; 12M: n=5; 18M: n=5) vs $Sirt1^{COIN/COIN}$ controls(3M: n=9; 12M: n=5; 18M: n=5)**.** (E) Representative images of spines from 3-,12- and 18-month-old female $Sirt1_{Syn}$-/- mice vs $Sirt1^{COIN/COIN}$ controls stained with Von Kossa**.** (F) BV/TV (%); (G) N.Ob/T.Ar (/mm^2); (H) BFR/BS (µm^3/µm^2/yr) and (I) Oc.S/BS (%) of 3-,12- and 18-month-old female $Sirt1_{Sert}$ -/- mice (3M: n=5; 12M: n=6; 18M: n=6) vs $Sirt1^{COIN/COIN}$ controls (3M: n=5; 12M: n=5; 18M: n=7)**.** (J) Representative images of spines from 3-, 12- and 18-month-old female $Sirt1_{Sert}$-/- mice vs $Sirt1^{COIN/COIN}$ controls stained with Von Kossa**.** (K) BV/TV (%); (L) N.Ob/T.Ar (/mm^2); (M) BFR/BS (µm^3/µm^2/yr) and (N) Oc.S/BS (%) of 3-and 12-month-old female $Sirt1_{Dbh}$ -/- mice (3M: n=6; 12M: n=7) vs $Sirt1^{COIN/COIN}$ controls(3M: n=5; 12M: n=6)**.** (O) Representative images of spines from 3- and 12-month-old female $Sirt1_{Dbh}$ -/- mice vs $Sirt1^{COIN/COIN}$ controls stained with Von Kossa. (P) BV/TV (fold to $Sirt1^{COIN/COIN}$ control) of male $Sirt1_{Syn}$ -/- mice (3M: n=9; 12M: n=6; 18M: n=5) vs female $Sirt1_{Syn}$ -/- mice (3M: n=8; 12M: n=5; 18M: n=5) at 3-,12- and 18-months. (Q) BV/TV (fold to $Sirt1^{COIN/COIN}$ control) of male $Sirt1_{Sert}$ -/- mice (3M: n=8; 12M: n=8; 18M: n=9) vs female $Sirt1_{Sert}$ -/- mice (3M: n=5; 12M: n=6; 18M: n=6) at 3-,12- and 18-months. . (R) BV/TV (fold to $Sirt1^{COIN/COIN}$ control) of male $Sirt1_{Dbh}$ -/- mice (3M: n=5; 12M: n=7) vs female $Sirt1_{Dbh}$ -/- mice (3M: n=6; 12M: n=7) at 3- and 12- months.

Data are presented as mean ± SEM. *: p<0.05 vs $Sirt1^{COIN/COIN}$ by Student's *t* test.

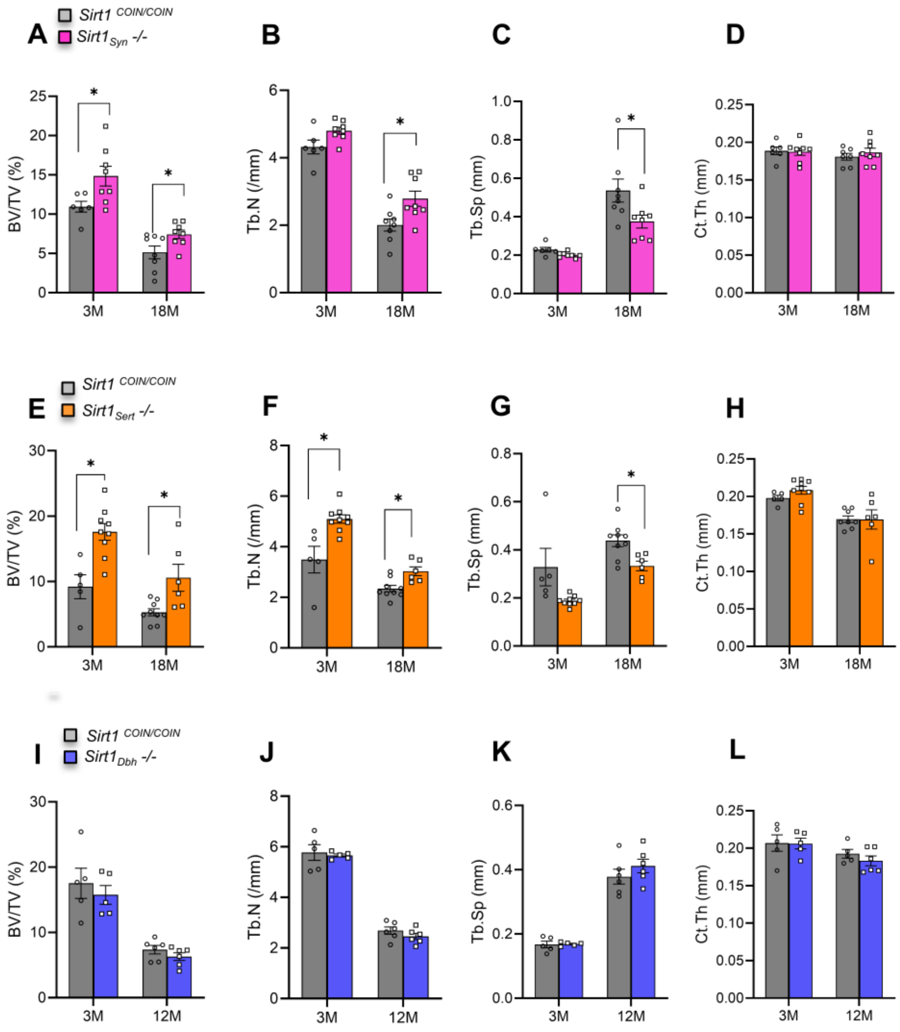

Supplemental Figure 4. Inactivation of *Sirt1* in serotonergic and *MAO-A*-expressing neurons, but not in the locus coeruleus, increases bone mass at proximal tibiae in male mice. (A) BV/TV (%); (B) Trabecular Number (Tb.N) (/mm); (C) Trabecular separation (Tb.Sp) (mm) and (D) Average cortical thickness (Ct.Th) (mm) of 3- and 18-month-old male $Sirt1_{Syn}$ -/- mice (3M: n=8; 18M: n=8) vs $Sirt1^{COIN/COIN}$ controls (3M: n=6; 18M: n=8)**.** (E) BV/TV (%); (F) Tb.N (/mm); (G) Tb.Sp (mm) and (H) Ct.Th (mm) of 3- and 18-month-old male $Sirt1_{Sert}$ -/- mice (3M: n=9; 18M: n=6) vs $Sirt1^{COIN/COIN}$ controls (3M: n=5; 18M: n=9)**.** (I) BV/TV (%); (J) Tb.N (/mm); (K) Tb.Sp (mm) and (L) Ct.Th (mm) of 3- and 12- month-old male $Sirt1_{Dbh}$-/- mice (3M: n=5; 12M: n=6) vs $Sirt1^{COIN/COIN}$ controls (3M: n=5; 12M: n=6)**.**
Data are presented as mean ± SEM. *: p<0.05 vs $Sirt1^{COIN/COIN}$ by Student's *t* test.

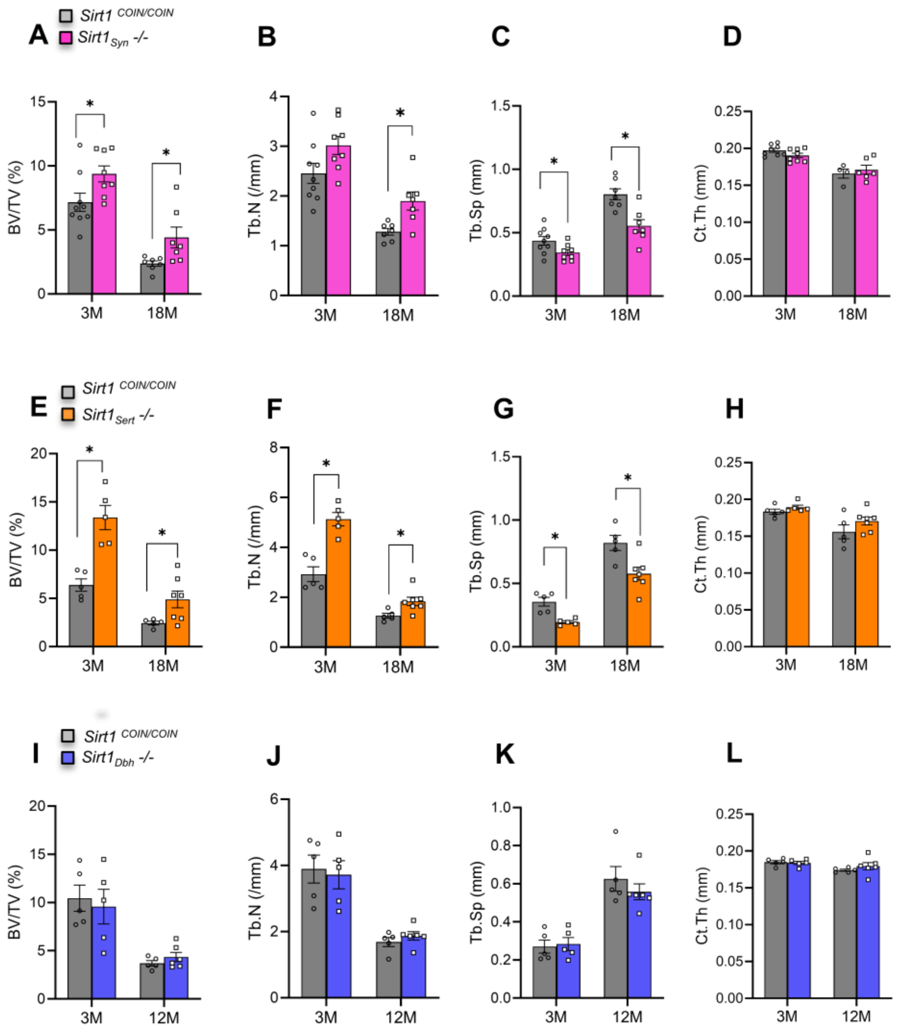

Supplemental Figure 5. Inactivation of *Sirt1* in serotonergic and *MAO-A*-expressing neurons, but not in the locus coeruleus, increases bone mass at proximal tibiae in female mice. (A) BV/TV (%); (B) Tb.N (/mm); (C) Tb.Sp (mm) and (D) Ct.Th (mm) of 3- and 18-month-old female $Sirt1_{Syn}$ -/- mice (3M: n=8; 18M: n=7) vs $Sirt1^{COIN/COIN}$ controls (3M: n=8; 18M: n=7)**.** (E) BV/TV (%); (F) Tb.N (/mm); (G) Tb.Sp (mm) and (H) Ct.Th (mm) of 3- and 18-month-old female $Sirt1_{Sert}$ -/- mice (3M: n=5; 18M: n=7) vs $Sirt1^{COIN/COIN}$ controls (3M: n=5; 18M: n=5)**.** (I) BV/TV (%); (J) Tb.N (/mm); (K) Tb.Sp (mm) and (L) Ct.Th (mm) of 3- and 12- month-old female $Sirt1_{Dbh}$-/- mice (3M: n=5; 12M: n=6) vs $Sirt1^{COIN/COIN}$ controls (3M: n=5; 12M: n=5)**.**
Data are presented as mean ± SEM. *: p<0.05 vs $Sirt1^{COIN/COIN}$ by Student's *t* test.

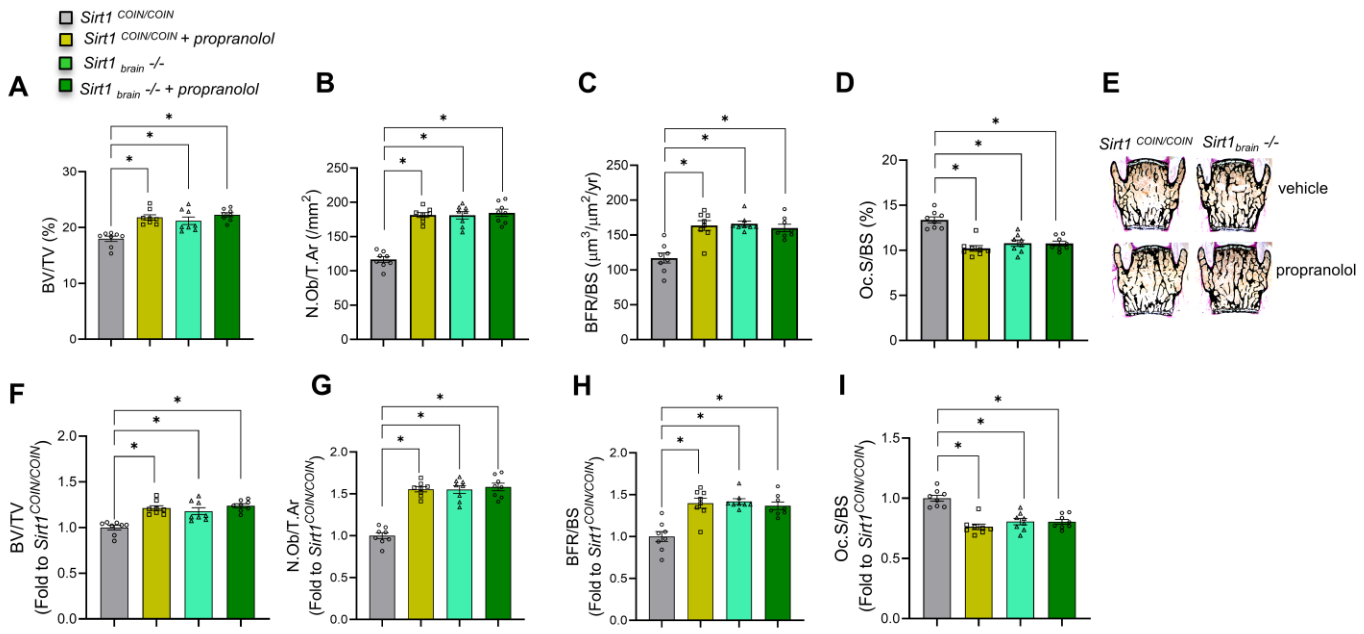

Supplemental Figure 6. Inhibition of SNS output in osteoblasts by propranolol treatment does not further increase bone mass in mice with inactivated *Sirt1* in the brain. (A) BV/TV (%); (B) N.Ob/T.Ar (/mm^2); (C) BFR/BS (µm^3/µm^2/yr) and (D) Oc.S/BS (%) of 3-month-old male $Sirt1^{COIN/COIN}$ mice treated with vehicle (n=8) vs $Sirt1^{COIN/COIN}$ mice treated with propranolol (n=8) vs Adeno-CMV-Cre i.c.v. injected $Sirt1^{COIN/COIN}$ ($Sirt1_{brain}$-/-) mice treated with vehicle (n=8) vs Adeno-CMV-Cre i.c.v. injected $Sirt1^{COIN/COIN}$ ($Sirt1_{brain}$-/-) mice treated with propranolol (n=8). (E) Representative images of spine from 3-month-old male $Sirt1^{COIN/COIN}$ mice treated with vehicle or propranolol and $Sirt1_{brain}$-/- mice treated with vehicle or propranolol stained with Von Kossa. (F) BV/TV (fold to $Sirt1^{COIN/COIN}$ control); (G) N.Ob/T.Ar (fold to $Sirt1^{COIN/COIN}$ control); (H) BFR/BS (fold to $Sirt1^{COIN/COIN}$ control) and (I) Oc.S/BS (fold to $Sirt1^{COIN/COIN}$ control) of 3-month-old male $Sirt1^{COIN/COIN}$ mice treated with vehicle (n=8) vs $Sirt1^{COIN/COIN}$ mice treated with propranolol (n=8) vs $Sirt1_{brain}$-/- mice treated with vehicle (n=8) vs $Sirt1_{brain}$-/- mice treated with propranolol (n=8).
Data are presented as mean ± SEM. *: $p<0.05$ vs $Sirt1^{COIN/COIN}$ by one-way ANOVA.

Supplemental Table 1. Primers for genotyping by PCR

	Sequence	
SirtTg F	5'-TGG AGG GGA TCA AGA GGT TGT TAA-3'	*TgSirt1*
SirtTg R	5'-CCA AGA AGA CAA TCT ATT TTC CAG-3'	
Sirt1 WT F	5'-GTT CAG CAA CAT CTC ATG ATT GG-3'	*Sirt1* $^{COIN/COIN}$
Sirt1 WT R	5'-GAA CAA AGG CTA CAT CAA GAG C-3'	
Sirt1 COIN F	5'-CAC TTT CTA CTC TGT TGA C-3'	
Sirt1 COIN R	5'-CCT TAC ATG TTT TAC TAG-3'	
Sirt Recomb F	5'-GTT TTC AGG GTG TTG TTT AG-3'	*Sirt1* rebombination
Sirt Recomb R	5'-CTG AAG CAC TGC ACG CCG TAG-3'	
oIMR1084	5'-GCG GTC TGG CAG TAA AAA CTA TC-3'	*Cre*
oIMR1085	5'-GTG AAA CAG CAT TGC TGT CAC TT-3'	
oIMR7338	5'-CTA GGC CAC AGA ATT GAA AGA TCT-3'	
oIMR7339	5'-GTA GGT GGA AAT TCT AGC ATC ATC C-3'	

Supplemental Table2. Primers for qPCR gene expression analysis

		Sequence
Runx2	Forward	5'-GCC GGG AAT GAT GAG AAC TA-3'
	Reverse	5'-GGA CCG TCC ACT GTC ACT TT-3'
Ocn	Forward	5'-CAG ACA AGT CCC ACA CAG CA-3'
	Reverse	5'-CTT GGC ATC TGT GAG GTC AG-3'
RankL	Forward	5'-AAG ATG CGA CGT ACT TTG GG-3'
	Reverse	5'-CGT GGG CCA TGT CTC TTA GT-3'
Opg	Forward	5'-GAA AGA CCT GCA AAT CGA GC-3'
	Reverse	5'-TTG TGA AGC TGT GCA GGA AC-3'
Ctsk	Forward	5'-ACG GAG GCA TTG ACT CTG AAG ATG-3'
	Reverse	5'-GGA AGC ACC AAC GAG AGG AGA AAT-3'
Ucp1	Forward	5'-AGG CTT CCA GTA CCA TTA GGT-3'
	Reverse	5'-CTG AGT GAG GCA AAG CTG ATT T-3'
Bmal-1	Forward	5'-CCA AGA AAG TAT GGA CAC AGA CAA A-3'
	Reverse	5'-GCA TTC TTG ATC CTT CCT TGG T-3'
Per-1	Forward	5'-ACC AGC GTG TCA TGA TGA CAT AC-3'
	Reverse	5'-CTC TCC CGG TCT TGC TTC AG-3'
Per-2	Forward	5'-ATG CTC GCC ATC CAC AAG A-3'
	Reverse	5'-GCG GAA TCG AAT GGG AGA AT-3'
Cry-1	Forward	5'-CTG GCG TGG AAG TCA TCG T-3'
	Reverse	5'-CTG TCC GCC ATT GAG TTC TAT G-3'
Tph2	Forward	5'-GTG ACC CTG AAT CCG CCT G-3'
	Reverse	5'-GGT GCC GTA CAT GAG GAC T-3'
MAOA	Forward	5'-TCT GTT GGA CAA AAA CTG CTC-3'
	Reverse	5'-ATT TGG CCA GAG CCA CCT A-3'
Dbh	Forward	5'-GAG GCG GCT TCC ATG TAC G-3'
	Reverse	5'-TCC AGG GGG ATG TGG TAG G-3'
Bche	Forward	5'-TCT ATG AGG CCA CCA TGA GGC TC-3'
	Reverse	5'-AGT GCC ACC TTG ACA GCA TCT GGA-3'
Sirt1	Forward	5'-CAG ACC CTC AAG CCA TGT TT-3'
	Reverse	5'-GAT CCT TTG GAT TCC TGC AA-3'
beta actin	Forward	5'-GAC CTC TAT GCC AAC ACA GT-3'
	Reverse	5'-AGT ACT TGC GCT CAG GAG GA-3'
